# Quality of life changes in cluster headache: Convergent validity, responsiveness, and interpretability of the Cluster Headache Quality of Life scale as a patient‐reported outcome measure

**DOI:** 10.1111/head.15083

**Published:** 2025-11-13

**Authors:** Willemijn C. Naber, Paulien J. van Tilborg, Roemer B. Brandt, Julia J. Jansen, Leopoldine A. Wilbrink, Wim M. Mulleners, Erkan Kurt, Willemijn Leen, Frank J. P. M. Huygen, Denise Bijlenga, Rolf Fronczek

**Affiliations:** ^1^ Department of Neurology Leiden University Medical Center (LUMC) Leiden The Netherlands; ^2^ Department of Neurology The Migraine Clinic Amsterdam The Netherlands; ^3^ Department of Neurology Canisius Wilhelmina Ziekenhuis Nijmegen The Netherlands; ^4^ Department of Pharmacy, Pharmacology and Toxicology Radboud University Medical Center Nijmegen The Netherlands; ^5^ Department of Neurology Zuyderland Medical Center Heerlen The Netherlands; ^6^ Department of Neurosurgery Radboud University Medical Center Nijmegen The Netherlands; ^7^ Department of Anesthesiology, Pain and Palliative Medicine Radboud University Medical Center Nijmegen The Netherlands; ^8^ Department of Anaesthesiology Erasmus Medical Center Rotterdam The Netherlands; ^9^ Stichting Epilepsie Instellingen Nederland (SEIN), Sleep‐Wake Center Heemstede The Netherlands

**Keywords:** cluster headache, Cluster Headache Quality of Life scale, patient‐reported outcome measure, quality of life

## Abstract

**Objective:**

Cluster headache (CH) significantly impacts patients' quality of life (QoL). We aim to validate the Cluster Headache Quality of Life scale (CHQ) for measuring QoL changes in patients with CH.

**Methods:**

In this multicenter, prospective, longitudinal psychometric validation study, participants, all with chronic CH (CCH), completed the CHQ at a 3‐monthly interval alongside headache diaries and general QoL questionnaires (36‐item Short Form [SF‐36], Hospital Anxiety and Depression Scale [HADS], and EuroQol 5 Dimensions [EQ‐5D]). CHQ's ability to measure changes was validated in three steps following COSMIN guidelines: (1) convergent validity, (2) responsiveness, and (3) interpretability. Baseline scores were used for step 1; change scores for steps 2 and 3 (Δ baseline—follow‐up). Twelve correlation hypotheses were formulated and tested for steps 1 and 2. Validity was rated by % rejected hypotheses (high: ≤25%, moderate: 26%–50%, poor: ≥50%). Data were collected in the Netherlands (Leiden, Nijmegen, Heerlen) between 9 December 2021 and 18 November 2024.

**Results:**

For step 1, 117 participants were included (*n* = 70 for SF‐36/HADS analyses, *n* = 42 for EQ‐5D analyses) and 82 were included for steps 2 and 3 (*n* = 48 for SF‐36/HADS analyses, *n* = 29 for EQ‐5D analyses). At baseline, overall QoL was poor (CHQ: 60.9 ± 23.1, SF‐36: 46.7 ± 19.9) and worse in participants with more intense and frequent CH attacks (>CHQ scores: *β* = 2.92 (95% confidence interval [CI] 0.91 to 4.93), *p* = 0.005; *β* = 0.27 (95% CI 0.02 to 0.53), *p* = 0.033). Convergent and responsiveness validity was high (≤25% hypotheses rejected). CHQ baseline scores correlated strongly with the HADS, SF‐36, and EQ‐5D (*ρ* = 0.68, *ρ* = −0.60, *ρ* = −0.52), but weakly with attack frequency (*ρ* = 0.27). Change scores correlated strongly with HADS and SF‐36 (*ρ* = 0.51, *ρ* = −0.56) and moderately with EQ‐5D (*ρ* = −0.38). Step 3 indicated ≤ −3.5 points change as clinically relevant improvement and ≥7.5 points as deterioration.

**Conclusion:**

The CHQ has a high validity to measure change in QoL. CH attack frequency influences QoL, but QoL is more strongly correlated with mental health and activity restrictions than attack frequency. Implementing the CHQ may improve understanding of disease burden, enabling more targeted treatment strategies and thus improving overall disease management.

## Plain Language Summary

People with cluster headache often experience significant disability and poor quality of life. We wanted to learn what factors most strongly influence quality of life for these patients and determine whether the Cluster Headache Quality of Life scale can reliably track changes over time as patients try new treatments. We found that mental health and ability to complete daily tasks affect quality of life more than the number of attacks a patient has, and that this survey can help doctors better understand the full impact of cluster headache and improve treatment decisions.

**Abbreviations**
CCH: chronic cluster headache
CH: cluster headache
CHQ: Cluster Headache Quality of Life scale
CI: confidence interval
COSMIN: COnsensus‐based Standards for the selection of health Measurement INstruments
ECH: episodic cluster headache
EQ‐5D: EuroQol 5 Dimensions
EQ‐5D‐5L: EuroQol 5 Dimensions 5 Levels
EQ VAS: EuroQol visual analogue scale
FAM: Fear Avoidance Model
GON: greater occipital nerve
HADS: Hospital Anxiety and Depression Scale
ICHD3: International Classification of Headache Disorders, third edition
IQR: interquartile range
LUMC: Leiden University Medical Center
METC‐LDD: ethical committee of the Leiden University Medical Center
MIC: minimally important change
ONS: occipital nerve stimulator
PROMs: patient‐reported outcome measures
QoL: quality of life
ROC: receiver‐operating characteristic
SD: standard deviation
SF‐36: 36‐item Short Form
VAS: visual analogue scale

## INTRODUCTION

Cluster headache (CH) is a primary headache characterized by unilateral, extremely painful, headache attacks.^1^ In episodic cluster headache (ECH, 80% of cases) the attacks occur in episodes lasting weeks or months, with remission intervals of months to years.^1^ Patients with chronic cluster headache (CCH, 20% of cases) have headache attacks with less than 3 consecutive months of remission per year.^2^ Due to the severity of the disease, CH can lead to significant disability and a decreased quality of life (QoL).^3^ During clinical or research follow‐up, the burden of disease is mainly assessed by symptom severity and—especially—attack frequency. However, as is highlighted by discrepancies between attack frequency and patient‐reported treatment satisfaction in clinical trial results, attack frequency alone does not fully explain the perceived disease burden.

In recent years, there has been a growing focus on patient‐reported outcome measures (PROMs). Reflecting this, the revised clinical trial guidelines for cluster headache recommend incorporating these measures as clinical trial endpoints.^4^ Previously, no CH‐specific questionnaires were available and QoL was measured with generic scales (e.g., 36‐itme Short Form [SF‐36]). However, recently, cluster headache‐specific QoL questionnaires, such as the Cluster Headache Quality of Life scale (CHQ), have been developed.^5^ When implemented, these questionnaires will improve our understanding of the specific QoL domains affected in CH and bridge the gap between objective symptom severity and subjective patient‐reported QoL. This will facilitate more accurate assessment of disease burden in both clinical practice and research evaluations.

To accurately assess QoL in CH, it is essential to verify the reliability and validity of this disease‐specific instrument for use by researchers and clinicians. The COSMIN (COnsensus‐based Standards for the selection of health Measurement INstruments) guidelines underline that validation should not only address scores at a single time point but also the sensitivity and accuracy of capturing change in scores over time.^6^, ^7^ Change scores are used to draw conclusions about the effect of treatment on QoL and patient improvement, therefore ensuring reliability of these scores is essential. Responsiveness is the term that is used to describe the ability of an instrument to detect changes over time in the construct (the intended concept to be measured, here QoL) that it measures.^7^

The CHQ is a short, easy to use questionnaire relating to patients' daily activities. The scale was developed in consultation with people with cluster headache and clinicians in 2016 and has subsequently been validated, translated, and implemented in various Dutch clinical studies.^5^, ^8^ The CHQ has been established as reliable and its construct validity was excellent.^5^, ^8^ For convergent validity (correlation between measures of related topics), it has been shown that CHQ scores correlate with those of the SF‐36 and the EuroQol 5 Dimensions (EQ‐5D) when comparing them at a single timepoint. However, correlation between changes in scores of these questionnaires and CHQ's correlation to attack frequency or intensity have not yet been studied.^5^, ^8^ Additionally, validation of the responsiveness of the CHQ—its “sensitivity to change”—and the minimally clinically relevant change in the CHQ have not yet been investigated.^5^

We aim to validate the ability of the CHQ to measure QoL changes in CH in three steps: (i) convergent validity, (ii) responsiveness, and (iii) interpretability of the CHQ. Convergent validity will be assessed by exploring the impact of, and correlation between, factors influencing QoL in chronic cluster headache. Secondly, we aim to examine the responsiveness of the CHQ to validate whether it can accurately detect changes in CH symptoms and QoL over time, and lastly we aim to define guidelines for interpreting changes in CHQ scores. Overall, validating the CHQ can provide valuable insights into the burden of CH and facilitate its implementation in clinical practice and clinical trials, ultimately improving disease management and research outcomes.

## METHOD

### Design and participants

In this multicenter, prospective, longitudinal psychometric validation study, participants, all with chronic cluster headache, were included who completed the CHQ as part of two ongoing Dutch prospective CCH cohorts (ONSFU cohort, NL75958.058.20; REGON cohort, NCT 05324748) via three tertiary referral centers (Leiden University Medical Center, Canisius Wilhelmina Hospital, Zuyderland Medical Center). In the REGON cohort, a double‐blinded randomized clinical trial is conducted in which the intervention consists of repeated greater occipital nerve (GON) injections with either methylprednisolone (80 mg) or placebo (NaCl 0.9%). In the ONSFU cohort, an observational prospective study, the intervention involves implantation of an occipital nerve stimulator (ONS). The current study focuses on changes in QoL, as measured by the CHQ, irrespective of the intervention. Efficacy of the respective interventions of the REGON and ONSFU will be published separately upon completion of these studies.

Inclusion criteria for the current study were: diagnosis of chronic cluster headache as defined by the International Classification of Headache Disorders, third edition (ICHD3) criteria^1^ (thus active cluster headache during both measurements), age ≥ 16 years, proficient in Dutch to complete the questionnaires and e‐diaries. For the *baseline* analysis, all participants with sufficient survey compliance and completion of one or more CHQ were included. For *change* analysis, participants needed to have completed the CHQ twice or more with an interval of approximately 3 months. When possible, CHQ measurements surrounding pre‐ and post‐intervention (baseline and follow‐up) were selected. If preintervention data were unavailable, the first subsequently completed measurement was used as baseline assessment. This ensures that enough “change” in patients' cluster headache symptoms could be expected during the 3‐month interval. All participants provided written informed consent for trial‐participation and a waiver for use of secondary data for this study. Ethical approval was provided by the Medical Research Ethics Committee of the Leiden University Medical Center (METC‐LDD of LUMC; REGON: P22.022;ONSFU: P21.004). Data were collected between 9 December 2021 and 18 November 2024.

In order to have adequate power to perform a responsiveness analysis, the COSMIN guideline recommends to include at least 30 patients. No formal statistical power calculation was conducted prior to the study. Instead, the sample size was determined by available data from our clinical cohort. We verified that our sample size met the minimum COSMIN requirements before proceeding with the analysis.

### Questionnaires

Participants completed the CHQ at 3‐monthly intervals. Headache diaries and QoL questionnaires that were completed during the same timeframe as the CHQ were collected. Both cohorts completed the CHQ and cluster headache diaries during their prospective follow‐up. The ONSFU cohort also completed the Hospital Anxiety and Depression Scale (HADS) and the 36‐item Short Form (SF‐36), where the REGON cohort completed the EQ‐5D 5 Levels (EQ‐5D‐5L) on top of the CHQ.

#### CHQ scale (CHQ)

The CHQ scale is a validated questionnaire that consists of 28 questions covering four domains related to QoL: “restriction of activities of daily living,” “impact on mood and interpersonal relationships,” “pain and anxiety,” and “lack of vitality” (Supporting Information S1).^5^, ^8^ Items are scored on a 5‐point Likert scale (Never = 0, Always = 4) with a minimum score of 0 and maximum score 112, where higher scores indicate poorer health related QoL. In addition, the CHQ also records the amount of cluster headache attacks in the previous month and a visual analogue scale (VAS) ranging from “not at all satisfied with life” to “very satisfied with life” is included. These last two items are not included in the total CHQ score.

#### CH diary

The CH diary is an electronic diary in which participants record the weekly attack frequency, duration, and intensity (11‐point Likert scales, ranging from ‘0 = No pain’ to ‘10= Worst pain imaginable’) and general well‐being (7‐point Likert scale, ranging from ‘0 = Very bad’ to ‘6 = Very well’).

#### HADS

The HADS is a validated questionnaire to screen for anxiety disorder and depression, that consists of 14 items that are answered on a 4‐point Likert scale (range 0–3).^9^ The subscales Depression and Anxiety are scored as the sum of the seven associated items with a score range of 0–21, with higher scores indicating more symptoms associated with anxiety and depression (cutoff scores are defined as: no (0–8), possible (8–10), or suspected (11–21) anxiety or depression disorder).

#### EQ‐5D

The EQ‐5D is a validated generic quality of life questionnaire that uses a descriptive system of five health domains (mobility, self‐care, usual activities, pain, and anxiety) and is scored on a 5‐point Likert scale ranging from no problem (1) to extreme problems (5).^10^ An index value can be derived that represents the health state ranging between −0.599 and 1 (1: best possible health state, 0 is death and negative values represent health states perceived as worse than dead).^11^ The EuroQol visual analogue scale (EQ VAS) scores general health on a scale from 0 (worst imaginable health state) to 100 (best imaginable health state). A change of 0.03 on the EQ‐5D index has been defined as the minimally important change (MIC).^12^, ^13^, ^14^

#### SF‐36

The SF‐36 is a generic QoL measurement with excellent reliability and validity that measures general health‐related QoL over the past 4 weeks.^15^ It measures functioning with eight subscales (Physical Functioning, Role Physical, Bodily Pain, General Health, Vitality, Social Functioning, Role Emotional, and Mental Health) and two component scores (Mental and Physical Component). All subscales and component scores are scored on a scale from 0 to 100 with higher scores indicating better QoL. A change of 3 points was defined as MIC.^15^, ^16^

### Validation analyses

The ability of the CHQ to measure QoL changes in CH was validated in three steps:

1. Convergent validity: the ability of the CHQ to measure QoL at *baseline* by evaluating correlation of the CHQ with other measurements.
2. Responsiveness validity: the ability of the CHQ to detect *change* in QoL over time (i.e., following treatment).
3. Interpretability: define guidelines for interpreting changes in CHQ scores by determining the MIC.

The recommendations for the reporting and validation of PROMS as stated by the COSMIN guidelines were followed.^6^, ^7^ For steps 1 and 2, a hypotheses‐driven correlation analysis was applied, as advised by the COSMIN guidelines in the absence of a gold standard for CH‐specific QoL outcomes.^6^ Specific hypotheses regarding correlations between CHQ, SF‐36, HADS, EQ‐5D, and headache diary outcomes were independently formulated by the authors (P.T., R.B., W.N.) and finalized in a consensus meeting, resulting in 12 hypotheses. Validity was considered high if 25% or less of the hypotheses were rejected, moderate if 26%–50% were rejected, and poor if more than 50% of the hypotheses were rejected, per recommendation by the COSMIN guidelines.^17^, ^18^ The hypothesis‐driven correlation analysis was applied twice: initially for correlations between *baseline* scores (step 1. convergent validation) and then for correlations between *changes* scores (step 2. responsiveness validity). A sensitivity analysis was performed to repeat correlation analysis of both steps stratified by cohort (ONSFU vs. REGON cohort) to address whether population heterogeneity has a significant effect.

### Step 1. Convergent validity

The hypotheses‐driven correlation analysis was applied to validate convergent validity and the hypotheses proposed about correlations between the *baseline* scores of CHQ, SF‐36, HADS, EQ‐5D, and headache diary measurements are presented in Table 1. Hypotheses 2–4 and 6–12 pertain to correlations of the score of the CHQ and other QoL measurements. Hypotheses 1 and 5 pertain to correlations of the CHQ and disease activity as measured by headache diaries. In general, a higher correlation was expected between the CHQ (subscales) and items that overlap due to similar wording and items that focus on mental health. Argumentation for individual hypotheses can be found in Supporting Information S2.

**TABLE 1:** Hypotheses for confirming convergent validity.

Hypotheses	Hypothesis confirmed or rejected	Comments
The CHQ total score has a *moderate* or weaker positive correlation (*ρ* ≤ 0.39) with the attack frequency.	Confirmed	*ρ* = 0.27 ONSFU *ρ* = 0.27 (✓), REGON *ρ* = 0.35 (✓)
2 The CHQ total score has a *strong* or stronger positive correlation (*ρ* ≥ 0.4) with the HADS total score.	Confirmed	*ρ* = 0.68 ONSFU *ρ* = 0.68 (✓), REGON *ρ* = N.A.
3 The CHQ total score has a *strong* or stronger negative correlation (*ρ* ≤ −0.4) with the SF‐36 total score	Confirmed	*ρ* = −0.60 ONSFU *ρ* = 0.60 (✓), REGON *ρ* = N.A.
4 The CHQ total score has a *moderate* or stronger negative correlation (*ρ* ≤ −0.3) with the EQ‐5D index.	Confirmed	*ρ* = −0.52 ONSFU *ρ* = N.A, REGON *ρ* = −0.52 (✓)
5 The CHQ total score correlates stronger with the EQ‐5D, SF‐36, and HADS, than with the attack frequency	Confirmed	Attack frequency: *ρ* = 0.27; EQ‐5D: *ρ* = −0.52; SF‐36: *ρ* = −0.60; HADS: *ρ* = 0.68. ONSFU ✓, REGON ✓ (Supporting Information S4)
6 None of the CHQ subscales (excluding the overall QoL question) have a *very strong* correlation (*ρ* ≥ 0.7/≤ − 0.7) with the subscales of the SF‐36, EQ‐5D, or the HADS.	Rejected	1/72 with *ρ* ≥ 0.7 or ≤−0.7; CHQ vitality & HADS depression: *ρ* = 0.70 ONSFU ✗, REGON ✓ (Supporting Information S4)
7 The overall QoL of the CHQ correlates stronger than the other CHQ subscales to the subscales of the SF‐36, EQ‐5D, and HADS.	Rejected	Overall QoL of the CHQ correlates mostly similar to the other subscales. ONSFU ✗, REGON ✗ (Supporting Information S4)
8 The CHQ mood subscale correlates stronger than the other subscales (excluding the overall QoL question) with subscales of the SF‐36, EQ‐5D, and the HADS.	Rejected	EQ5D: in 4/5 strongest correlation; SF‐36: in 1/8 strongest correlation; HADS: in 0/3 strongest correlation; ONSFU ✗, REGON ✗ (Supporting Information S4)
9 At least 50% of CHQ subscales have a *moderate* or weaker negative correlation (*ρ* ≥ −0.39) with the SF‐36 physical functioning subscale	Confirmed	2/4 of CHQ subscales correlate ≥ −0.39 with the SF‐36 physical functioning subscale. ONSFU ✓, REGON N.A.
10 The majority of CHQ subscales have a *moderate* or weaker negative correlation (*ρ* ≥ −0.39) with the SF‐36 general health subscale	Confirmed	3/4 of CHQ subscales correlate ≥ −0.39 with the SF‐36 general health subscale. ONSFU ✓, REGON N.A. (Supporting Information S4)
11 The majority of CHQ subscales have a *weak* or weaker positive correlation (*ρ* ≤ 0.29) with the EQ‐5D mobility subscale	Confirmed	4/4 of CHQ subscales correlate ≤0.29 with the EQ‐5D mobility subscale. ONSFU N.A, REGON ✓(Supporting Information S4)
12 The majority of CHQ subscales correlate at least negative *moderate* or stronger (*ρ* ≤ −0.30) with the SF‐36 mental health subscale	Confirmed	4/4 of CHQ subscales correlate ≤ −0.30 with the SF‐36 mental health subscale. ONSFU ✓, REGON N.A. (Supporting Information S4)
Total number of hypotheses that were rejected	3/12	
Percentage of hypotheses that were rejected	25%	Validity: **High**: ≤ 25% rejected (≤3/12); Moderate: 26%–50% rejected (4–6/12); Poor: ≥ 50% rejected (≥7/12)

*Note*: Specific hypotheses were proposed about correlations between the *baseline* scores of CHQ, SF‐36, HADS, EQ‐5D, and diary measurements before correlation analysis, as advised by the COSMIN guidelines.^6^ Validity was considered high if 25% or less of the hypotheses were rejected, moderate if 26%–50% were rejected and poor if more than 50% of the hypotheses were rejected. Spearman correlation interpretation: Very Strong, *ρ* ≥ 0.7; Strong, 0.4 ≤ *ρ* < 0.7; Moderate, 0.3 ≤ *ρ* < 0.4; Weak, 0.2 < *ρ* ≤ 0.3; No or negligible relationship, 0 ≤ *ρ* < 0.2. ✓ Hypothesis confirmed in the sensitivity analysis. **✗** Hypothesis not confirmed. Extended argumentation for each hypothesis can be found in Supporting Information S2.

Impact of baseline characteristics on the total score of the CHQ were explored using linear regression analysis.

### Step 2. Responsiveness validity

*Change* scores for the CHQ, SF‐36, HADS, EQ5D, and headache diary measurements were calculated as the difference between baseline and 3 months follow‐up assessments.

A second hypotheses‐driven correlation analysis was used to validate responsiveness. New hypotheses, describing expected correlations between *change* scores across instruments, were postulated after the *baseline* analysis. Hypotheses 1–3 and 6–12 concerned correlations between changes in CHQ scores and other QoL measurements (Table 2), whereas hypotheses 4 and 5 concerned correlations between changes in CHQ scores and headache diary outcomes.

**TABLE 2:** Hypotheses for responsiveness validity.

Hypotheses	Hypothesis confirmed or rejected	Comments
The change on the CHQ total score has a *strong* or stronger positive correlation (*ρ* ≥ 0.4) with change on the HADS total score.	Confirmed	*ρ* = 0.51 ONSFU *ρ* = 0.51 (✓), REGON *ρ* = N.A.
2 The change on the CHQ total score has a *strong* or stronger negative correlation (*ρ* ≤ −0.4) with change on the SF‐36 total score	Confirmed	*ρ* = −0.56 ONSFU *ρ* = −0.56 (✓), REGON *ρ* = N.A.
3 The change on the CHQ total score has a *moderate* or stronger negative correlation (*ρ* ≤ −0.3) with the change on the EQ‐5D index.	Confirmed	*ρ* = −0.38 ONSFU *ρ* = N.A., REGON *ρ* = −0.38 (✓)
4 The correlation between the change scores of the diary attack frequency and the CHQ total score is stronger than the correlation between the baseline scores of the total CHQ and the diary attack frequency (*ρ* > 0.27)	Confirmed	*ρ* = 0.36 ONSFU *ρ* = 0.28 (✓), REGON *ρ* = 0.47 (✓)
5 The change on the CHQ total score correlates stronger with the change on the SF‐36 and HADS, than with the change on the weekly attack frequency (diary)	Confirmed	*ρ* = −0.56 & *ρ* = 0.51 is stronger than *ρ* = 0.36 ONSFU *ρ* = −0.56 & 0.51 vs. *ρ* = 0.36 (✓) REGON *ρ* = N.A.
6 The majority of change scores of the CHQ subscales have a *weak* or weaker positive correlation (*ρ* ≤ 0.29) with the change on the EQ‐5D mobility subscale	Confirmed	3/4 have a *ρ* ≤ 0.29, only pain and anxiety has *ρ* = 0.31 ONSFU N.A., REGON ✓ (Supporting Information S5)
7 The majority of change scores of the CHQ subscales correlate at least negative *moderate* or stronger (*ρ* ≤ −0.30) with the change on the SF‐36 mental component subscale	Confirmed	4/4 have a *ρ* ≤ −0.30 ONSFU ✓, REGON N.A. (Supporting Information S5)
8 The change score of the CHQ monthly attack frequency has a *moderate* or stronger negative (*ρ* ≤ −0.30) correlation with the change of the SF‐36 physical component subscale	Confirmed	*ρ* = −0.30 ONSFU *ρ* = −0.30 (✓), REGON *ρ* = N.A.
9 The change score of the CHQ vitality has a *strong* or stronger positive correlation (*ρ* ≥ 0.40) with the change of the HADS total score	Confirmed	*ρ* = 0.42 ONSFU *ρ* = −0.42 (✓), REGON *ρ* = N.A.
10 The change score of the CHQ pain and anxiety has a *moderate* or weaker positive correlation (*ρ* ≤ 0.39) with the majority of the change scores of the EQ‐5D subscales	Confirmed	4/5, only EQ‐5D usual activities has a stronger correlation: *ρ* = 0.50 ONSFU N.A., REGON ✓ (Supporting Information S5)
11 The change score of the CHQ restriction of activities has a *strong* or stronger negative correlation (*ρ* ≤ − 0.40) with the change of the SF‐36 social functioning	Confirmed	*ρ* = −0.48 ONSFU *ρ* = −0.48 (✓), REGON *ρ* = N.A.
12 The change score of the CHQ vitality has a *strong* or stronger negative correlation (*ρ* ≤ −0.40) with the change of the SF‐36 vitality	Confirmed	*ρ* = −0.59 ONSFU *ρ* = −0.59 (✓), REGON *ρ* = N.A.
Total number of hypotheses that were rejected	0/12 rejected	
Percentage of hypotheses that were rejected	0%	Validity: **High**: ≤25% rejected (≤3/12); Moderate: 26%–50% rejected (4–6/12); Poor: ≥50% rejected (≥7/12)

*Note*: Specific hypotheses were proposed about correlations between the *change* scores of CHQ, SF‐36, HADS, EQ‐5D, and diary measurements before correlation analysis, as advised by the COSMIN guidelines.^6^ Validity was considered high if 25% or less of the hypotheses were rejected, moderate if 26%–50% were rejected, and poor if more than 50% of the hypotheses were rejected. Spearman correlation interpretation: Very Strong, *ρ* ≥ 0.7; Strong, 0.4 ≤ *ρ* < 0.7; Moderate, 0.3 ≤ *ρ* < 0.4; Weak, 0.2 < *ρ* ≤ 0.3; No or negligible relationship, 0 ≤ *ρ* < 0.2. ✓ Hypothesis confirmed in the sensitivity analysis. ✗ Hypothesis not confirmed. Extended argumentation for each hypothesis can be found in Supporting Information S3.

A stronger correlation was expected between change scores of overlapping domains, particularly those related to mental health or those demonstrating strong baseline correlations. Argumentation for individual hypotheses can be found in Supporting Information S3. Two secondary analyses explored these correlations (i) in a subpopulation of participants with impaired baseline QoL (SF‐36 score < 80 or EQ‐5D index <0.85, cutoff based normative data^11^, ^15^) and (ii) using relative rather than absolute change scores.

### Step 3. Interpretability

We defined guidelines for interpreting changes in CHQ scores by determining the MIC. For this, we first described the proportion of participants who had an important change in their QoL. Participants were classified in three groups (deteriorated, stable, improved) using anchors: a change of ≥3 points in SF‐36 score or ≥0.03 points in EQ‐5D index indicated improvement or deterioration, respectively.^12^, ^13^, ^14^, ^15^, ^16^ Participants whose QoL change fell between −0.03 and +0.03 points (EQ‐5D) or −3 and +3 points (SF‐36), respectively, were classified as stable. Each participant contributed data from only one instrument (SF‐36 or EQ‐5D), as the cohorts were distinct and nonoverlapping (REGON: SF‐36; ONSFU: EQ‐5D), thereby avoiding any ambiguity. The sum of participants in the “deteriorated” and “improved” groups represented the proportion of participants with a clinically significant changed QoL.

The visual anchor‐based MIC distribution method was used to determine the MIC of the CHQ as proposed by the COSMIN guidelines.^6^, ^19^ First, the population was grouped based on the previously described anchors. Second, the distribution of change scores of each group was plotted using proportional frequencies to ensure equal areas under the curves for the three groups. Lastly, the optimal receiver‐operating characteristic (ROC) cutoff point that led to the least misclassified participants was determined based on the value where [1−sensitivity] + [1−specificity] is smallest; this ROC cutoff point was the MIC value. Separate MIC values were calculated for “improvement” and “deterioration,” as these scores are known to differ, in accordance with COSMIN guidelines.

### Statistical analysis

All descriptive data were checked visually for distribution. They are presented as number (percentage) for categorical variables, mean ± standard deviation (SD) for symmetrically distributed continuous data, and median (interquartile range [IQR]) for skewed continuous data. Wilcoxon rank‐sum test and chi‐squared tests were used to compare population characteristics. Spearman's correlation was used to determine correlations between outcomes measures given the ordinal nature of the questionnaires (CHQ, HADS, SF‐36, EQ‐5D, and headache diary). The strength of Spearman's correlations was interpreted based on the absolute value of *ρ* (|*ρ*|): very strong, |*ρ*| ≥ 0.7; strong, 0.4 ≤ |*ρ*| < 0.7; moderate, 0.3 ≤ |*ρ*| < 0.4; weak, 0.2 < |*ρ*| ≤ 0.3; no or negligible relationship, 0 ≤ |*ρ*| < 0.2.^20^

The strength of Spearman's correlations was interpreted based on the absolute value of *ρ* (|*ρ*|): very strong (≥0.7), strong (0.4–0.69), moderate (0.3–0.39), weak (0.2–0.29), and negligible (<0.2).

The assumptions of linear regression—linearity, normality of residuals, and homoscedasticity—were evaluated using diagnostic plots from R (e.g., Q–Q plots and residualsversusfitted plots) generated for the model.

No missing data are expected for CHQ outcomes, as completion of the CHQ was part of the inclusion criteria. For the other patient‐reported outcome measures, missing data were handled using a complete‐case approach: patients with missing data for a specific instrument were excluded from the corresponding analyses.

Two‐tailed *p* values less than 0.05 were considered statistically significant. All analyses were performed using R (R Foundation for Statistical Computing, Vienna, Austria), version 4.3.1 in the RStudio Build 2023.6.1.524 environment (Posit PBC, Boston, MA, USA), using package *pROC* version 1.18.5.

## RESULTS

### Participants

**FIGURE 1:**
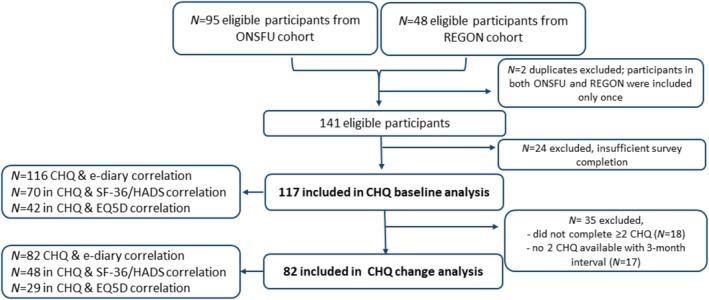
Study flowchart. In total 141 eligible participants were identified from two prospective CH cohorts (REGON and ONSFU cohort). Of those, 117 were included in the *baseline* analysis, and 82 met inclusion criteria for the *change* analysis. Participants were excluded due to insufficient survey completion at *baseline* (*N* = 24) or lack of 2 CHQs completed with an 3‐month interval for the *change* analysis (*N* = 35). For the CHQ baseline analyses, e‐diary data were missing in 1 of 117 participants, and 5 of 75 participants had incomplete SF‐36 and/or HADS data, while EQ‐5D data were complete (0/42 missing). For the CHQ change analyses, no missing data were observed for e‐diary or EQ‐5D outcomes; however, 5 of 53 ONSFU participants had missing SF‐36/HADS data, corresponding to the same individuals with missing baseline data. [Color figure can be viewed at wileyonlinelibrary.com]

A total of 141 eligible participants were identified in the prospective cluster headache cohorts. Of these, 117 were included in the *baseline* analysis to assess convergent validity (*n* = 70 for SF‐36/HADS analyses, *n* = 42 for EQ‐5D analyses); and 82 met the inclusion criteria for the *change* analysis determining responsiveness and interpretability (*n* = 48 for SF‐36/HADS analyses, *n* = 29 for EQ‐5D analyses, Figure 1). Participants were excluded due to insufficient survey completion at *baseline* (*n* = 24) or lack of 2 CHQs completed within a 3‐month interval for the *change* analysis (*n* = 35). Overall, missing data were limited. For the CHQ baseline analyses, e‐diary data were missing in 1 of 117 participants, and 5 of 75 participants had incomplete SF‐36 and/or HADS data, while EQ‐5D data were complete (0/42 missing). For the CHQ change analyses, no missing data were observed for e‐diary or EQ‐5D outcomes; however, 5 of 53 ONSFU participants had missing SF‐36/HADS data, corresponding to the same individuals with missing baseline data.

Participants were on average 47.0 (IQR: 34.0 to 57.0) years old and the male‐to‐female ratio was 3:2 (Table 3). No differences between the included and excluded participants were observed (age, 47.0 vs. 49.0 years; sex, 59% vs. 50% males; all *p* > 0.050). Participants from the ONSFU cohort were comparable to those of the REGON cohort (i.e., age, 47.0 vs. 49.0 years; sex, 56 vs. 64% males; all *p* > 0.050).

**TABLE 3:** Baseline and change analysis population characteristics, stratified by total population, ONSFU, and REGON cohorts.

Parameter	Baseline population	ONSFU cohort	REGON cohort	Change population	ONSFU cohort	REGON cohort
	Total (*N* = 117)	(*N* = 75)	(*N* = 42)	Total (*N* = 82)	(*N* = 53)	(*N* = 29)
Age, years	47.0 (34.0–57.0)	47.0 (34.5–57.5)	49.0 (34.5–54.0)	48 (35; 57)	47.0 (35.0–59.0)	49.0 (36.0–52.0)
Male	69 (59%)	42 (56%)	27 (64%)	50 (61%)	32 (60%)	18 (62%)
Years since diagnosis	12.0 (7.0–21.0)	13.0 (8.0–21.5)	9.0 (6.0–17.5)	11.0 (6.0–21.0)	16.0 (8.0–24.0)	8.0 (6.0–18.0)
Smoker	56 (49%)	33 (45%)	23 (55%)	39 (49%)	23 (45%)	16 (55%)
Comorbid Headache	21 (18%)	11 (15%)	10 (24%)	16 (20%)	8 (15%)	8 (28%)
Attack frequency, weekly	12.0 (4.4–25.1)	11.8 (3.9–28.2)	12.0 (7.8–20.3)	11.8 (4.0–25.3)	11.7 (3.8–28.3)	12.0 (8.3–18.0)
Attack intensity, scale 0–10	7.4 (6.0–8.7)	8.0 (6.0–9.5)	7.0 (6.2–7.7)	7.3 (6.0–8.5)	7.3 (6.0–9.0)	7.2 (6.3–8.0)
Attack duration, minutes	30.0 (18.3–60.0)	30.0 (20.0–61.7)	28.3 (18.3–60.0)	28.3 (18.3–60.0)	28.3 (18.3–61.7)	29.3 (18.3–60.0)
CHQ baseline score	60.8 (±23.3)	64.1 (±24.2)	54.9 (±20.4)	55.8 (±23.3)	58.2 (±24.6)	51.6 (±20.2)
*Source*						
ONSFU	75 (64%)	75 (100%)	0 (0%)	53 (65%)	53 (100%)	0 (0%)
REGON	42 (36%)	0 (0%)	42 (100%)	29 (35%)	0 (0%)	29 (100%)

*Note*: Population characteristics of the *baseline* population (*all eligible patient with at least 1 CHQ at baseline*), and *change* population (*all eligible patients with at least 2 CHQs with an interval of ±3 months in‐between*). For both the *baseline* and *change* population, data are stratified for the total population and the different treatment cohorts (ONSFU: Occipital nerve stimulation observational study, REGON: repeated GON injection clinical trial). Descriptives are depicted as mean (±SD) median (IQR) or number (percentage).

Abbreviation: NRS, numeric rating scale.

### Step 1. Convergent validity

#### Baseline QoL scores

**FIGURE 2:**
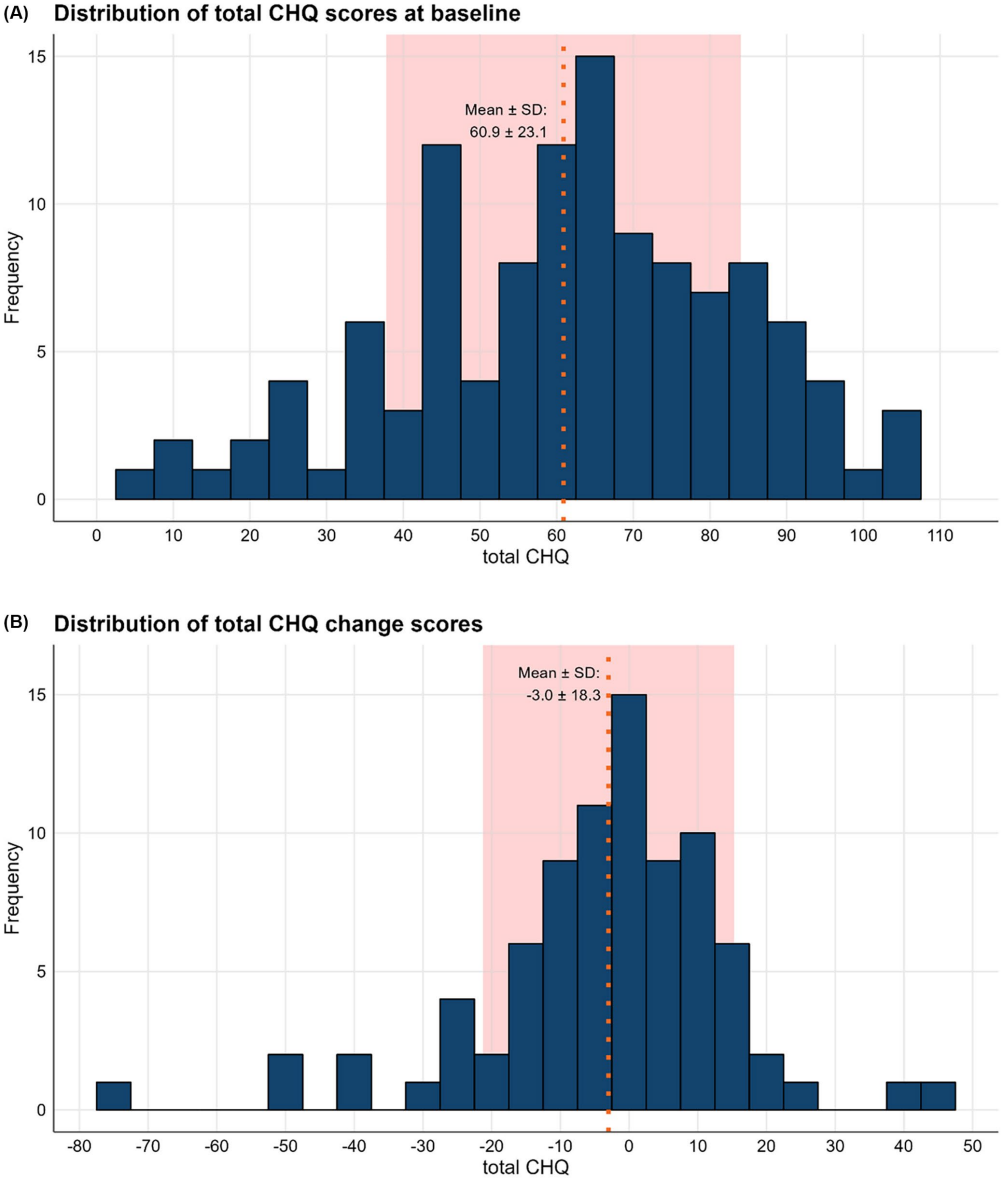
Distribution of total CHQ baseline and change scores. Histograms depicting the distribution of (A) baseline scores and (B) change scores (Δ baseline—follow‐up) of the total CHQ. The orange dotted line and background depict the mean and SD range. [Color figure can be viewed at wileyonlinelibrary.com]

All average *baseline* scores for the CHQ, SF‐36, EQ‐5D, and headache diaries are presented in Table 4. The mean total CHQ score was 60.9 ± 23.1 (SD) and CHQ score distribution did not show floor or ceiling effects (Figure 2). The median weekly attack frequency was 12.0 (IQR: 4.4 to 25.1). The mean EQ‐5D index was 0.61 ± 0.23 (SD), with Anxiety (2.1 ± 1.0 (SD)), Usual activities (2.3 ± 1.1), and Pain (3.1 ± 1.1) being the most affected domains. The mean SF‐36 total score was 46.7 ± 19.9 (SD), with Vitality (36.0 ± 21.6 (SD), Role physical (25.7 ± 39.3), Social functioning (20.8 ± 14.6), and Pain (16.1 ± 14.5) being the most affected subdomains. The mean HADS total score was 16.5 ± 9.5 (SD) with similarly high scores for the Anxiety and Depression subdomains (8.1 ± 4.9 and 8.5 ± 5.4, respectively).

**TABLE 4:** Average baseline and change scores of the CHQ, headache diary, EQ‐5D, the SF‐36, and the HADS.

Parameter	Baseline	Change	Directional^a^	Improved QoL group	Deteriorated QoL group
		scores			
	scores	Non‐directional^a^		scores	scores
CHQ	*N* = 117	*N* = 82	*N* = 82	*N* = 36	*N* = 22
Total	60.9 ± 23.1	Δ8.0 (3.0; 16.0)	Δ −3.2 ± 18.3	Δ −10.9 ± 21.5	Δ 6.7 ± 11.4
Restrictions of activities	21.9 ± 8.8	Δ4.0 (2.0; 7.0)	Δ −1.9 ± 7.4	Δ −5.3 ± 8.5	Δ 2.2 ± 5.0
Impact on mood and relationships	21.0 ± 10.3	Δ4.0 (2.0; 7.0)	Δ −0.3 ± 7.3	Δ −2.5 ± 8.0	Δ 3.1 ± 6.6
Pain and anxiety	5.4 ± 2.0	Δ2.0 (1.0; 3.0)	Δ −0.6 ± 2.1	Δ −1.1 ± 2.4	Δ 0.1 ± 1.5
Lack of vitality	12.6 ± 4.3	Δ2.0 (1.0; 3.0)	Δ −0.5 ± 4.1	Δ −2.1 ± 4.6	Δ 1.4 ± 3.0
Overall satisfaction, scale 0–10	5.4 ± 2.1	Δ1.0 (0.0; 2.0)	Δ 0.1 ± 1.6	Δ 0.7 ± 1.6	Δ −0.9 ± 1.3
Headache diary	*N = 116*	*N = 82*	*N = 82*	*N = 36*	*N = 22*
Attack frequency	12.0 (4.4; 25.1)	Δ3.6 (0.7;9.0)	Δ −1.1 ± 9.1	Δ −3.1 ± 7.7	Δ 2.7 ± 12.8
Attack intensity, scale 1–10	7.4 (6.0; 8.7)	Δ0.8 (0.2;1.5)	Δ −0.2 ± 1.9	Δ −0.9 ± 1.9	Δ 0.8 ± 1.9
Attack duration, minutes	30.0 (18.3; 60.0)	Δ6.5 (1.0;15.0)	Δ −7.3 ± 46.6	Δ −11.9 ± 55.3	Δ 2.7 ± 21.2
Overall wellbeing, scale 0–6	2.9 ± 1.2	Δ0.8 (0.2;1.2)	Δ 0.1 ± 1.2	Δ 0.7 ± 1.2	Δ −0.6 ± 0.9
EQ‐5D^b^	*N* = 42	*N* = 29	*N* = 29	*N* = 17	*N* = 7
VAS	59.3 ± 20.1	Δ17.0 (8.0; 25.0)	Δ 3.5 ± 20.6	Δ 9.8 ± 22.2	Δ−10.7 ± 9.1
Index	0.61 ± 0.23	Δ0.18 (0.04; 0.30)	Δ 0.1 ± 0.2	Δ 0.26 ± 0.17	Δ −0.14 ± 0.10
Mobility	1.3 ± 0.7	Δ0.0 (0.0; 0.0)	Δ −0.1 ± 0.6	Δ −0.1 ± 0.6	Δ 0.0 ± 0.6
Self‐care	1.1 ± 0.4	Δ0.0 (0.0; 0.0)	Δ −0.1 ± 0.3	Δ −0.1 ± 0.2	Δ −0.1 ± 0.4
Usual activities	2.3 ± 1.1	Δ1.0 (0.0; 1.0)	Δ −0.3 ± 1.1	Δ −0.6 ± 1.1	Δ 0.4 ± 0.8
Anxiety	2.1 ± 1.0	Δ1.0 (0.0; 1.0)	Δ −0.4 ± 1.0	Δ −1.0 ± 0.6	Δ 0.4 ± 1.1
Pain	3.1 ± 1.1	Δ1.0 (0.0; 1.0)	Δ −0.7 ± 1.2	Δ −1.3 ± 1.1	Δ 0.4 ± 0.8
SF‐36^b^	*N* = 70	*N* = 48	*N* = 48	*N* = 19	*N* = 15
Total	46.7 ± 19.9	Δ7.9 (2.6; 15.2)	Δ 0.1 ± 14.4	Δ 12.1 ± 7.8	Δ −16.4 ± 11.1
Physical component	50.0. ± 23.8	Δ5.4 (2.8; 14.6)	Δ −1.0 ± 14.9	Δ 10.3 ± 9.9	Δ −16.1 ± 13.3
Mental component	44.6 ± 21.2	Δ10.0 (2.9;20.0)	Δ 0.9 ± 18.3	Δ 14.1 ± 15.2	Δ −17.7 ± 14.6
Physical functioning	66.5 ± 25.8	Δ5.0 (5.0; 15.0)	Δ −1.4 ± 12.2	Δ 6.7 ± 11.4	Δ −11.2 ± 10.0
Role physical	25.7 ± 39.3	Δ0.0 (0.0; 28.1)	Δ −1.1 ± 40.1	Δ 19.7 ± 31.0	Δ −31.7 ± 47.7
Role emotional	50.2 ± 44.7	Δ16.7 (0.0;66.7)	Δ 1.1 ± 48.7	Δ 28.9 ± 35.9	Δ −46.7 ± 46.8
Vitality	36.0 ± 21.6	Δ5.0 (5.0; 11.3)	Δ −0.2 ± 15.1	Δ 7.89 ± 15.3	Δ −8.8 ± 14.2
Mental health	57.7 ± 21.9	Δ8.0 (4.0, 16.0)	Δ 2.0 ± 18.6	Δ 11.9 ± 20.1	Δ −10.0 ± 16.9
Social functioning	20.8 ± 14.6	Δ1.0 (0.0; 13.0)	Δ 0.2 ± 14.2	Δ 9.5 ± 13.5	Δ −11.1 ± 12.5
Pain	16.1 ± 14.5	Δ7.6 (0.0, 13.0)	Δ 1.3 ± 12.7	Δ 9.1 ± 12.3	Δ −9.6 ± 10.3
General health	41.2 ± 23.5	Δ5.0 (2.5, 15.0)	Δ 0.9 ± 13.3	Δ 14.1 ± 15.2	Δ −6.7 ± 12.5
HADS^b^	*N* = 70	*N* = 48	*N* = 48	*N* = 19	*N* = 15
Total	16.5 ± 9.5	Δ4.0 (2.0; 7.0)	Δ −0.4 ± 7.6	Δ −4.5 ± 7.3	Δ 5.3 ± 7.5
Anxiety	8.1 ± 4.9	Δ2.0 (1.0; 3.6)	Δ −0.6 ± 4.1	Δ −2.3 ± 4.2	Δ 2.1 ± 4.4
Depression	8.5 ± 5.4	Δ2.0 (1.0; 4.0)	Δ 0.2 ± 4.1	Δ −2.3 ± 3.7	Δ 3.2 ± 3.8

*Note*: Average baseline and change scores of the CHQ, Headache diary, EQ‐5D, the SF‐36, and the HADS. Data are either depicted as mean ± SD or as median (IQR).

^a^ Overall change scores are depicted as non‐directional Δ score, meaning this score is irrelative of this is a – or + score (e.g. change score of −1 is depicted as Δ1 and change score + 1 is also Δ1), and directional Δ score. The improved QoL group consists of all participants with either Δ +3 SF‐36 or Δ +0.03 EQ‐5D index. The deteriorated QoL group consists of all participants with either Δ −3 SF‐36 or Δ −0.03 EQ‐5D index. Change scores in these groups are the directional changes.

^b^ The EQ‐5D was completed only by the REGON cohort (*N* = 42 at baseline, *N* = 29 at change analysis). The SF‐36 and HADS were completed exclusively by the ONSFU cohort (*N* = 70 at baseline, *N* = 48 at change analysis).

#### Influence of patient characteristics on the CHQ

The linear regression showed that participants with higher attack intensity and frequency had poorer QoL, reflected by higher CHQ scores (*β* = 2.92 (95% CI 0.91 to 4.93), *p* = 0.005; *β* = 0.27 (95% CI 0.02 to 0.53), *p* = 0.033; Table 5). Sex was not significantly associated with baseline CHQ scores (*β* = 2.47 (95% CI −5.50 to 10.45), *p* = 0.540).

**TABLE 5:** Influence of baseline characteristics on the total score of the CHQ at baseline.

Parameter	*β*	95% CI	*p*‐Value
Age, years	−0.17	−0.50 to 0.12	0.222
Sex, female	2.47	−5.50 to 10.45	0.540
Years since diagnosis	−0.001	−0.020 to 0.017	0.888
Comorbid headache	−3.40	−12.80 to 6.00	0.474
Attack frequency, weekly	0.27	0.02–0.53	**0.033** *
Attack intensity, scale 0–10	2.92	0.91–4.93	**0.005** **
Attack duration, minutes	0.01	−0.07 to 0.08	0.871

*Note*: Results of linear regression to model the influence of baseline characteristics on the total score of the CHQ at baseline. These values were in bold because they were statistically significant.

* *p* < 0.05.

** *p* < 0.01.

#### Hypotheses‐driven correlation analysis

**FIGURE 3:**
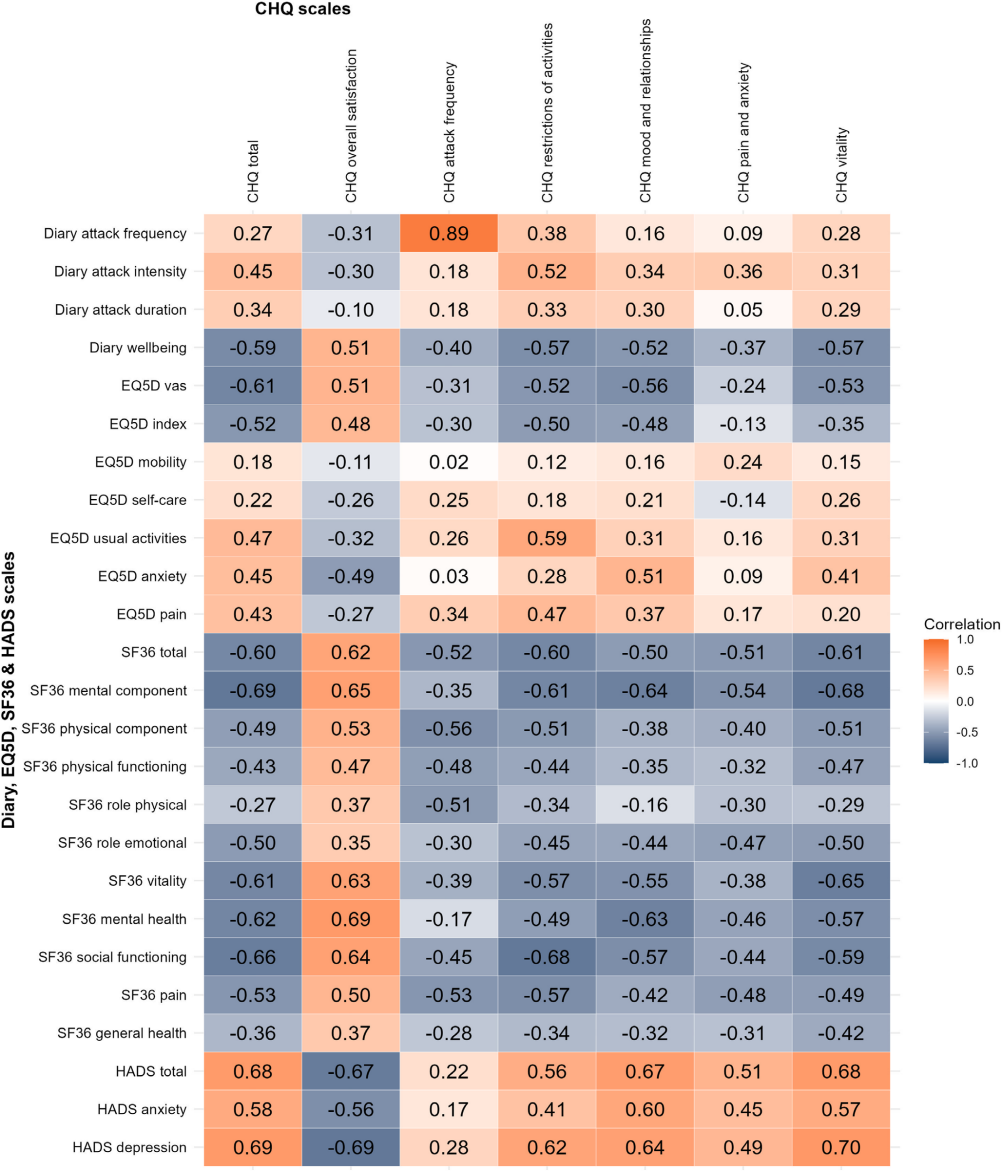
Heatmap depicting correlation coefficients of baseline CHQ scores with other patient reported outcomes. Heatmap depicting the spearman correlation coefficients of the baseline scores of the CHQ scales and other quality of life measurements (SF‐36, HADS, and EQ‐5D) or diary measurements. A darker color (either orange or blue) is associated with a stronger correlation. Spearman correlation interpretation: Very Strong, *ρ* ≥ 0.7; Strong, 0.4 ≤ *ρ* < 0.7; Moderate, 0.3 ≤ *ρ* < 0.4; Weak, 0.2 < *ρ* ≤ 0.3; No or negligible relationship, 0 ≤ *ρ* < 0.2. The CHQ and e‐diary were completed by all participants, the SF‐36/HADS only by the ONSFU cohort, and the EQ‐5D only by REGON participants (*N* = 117 participants in total, *n* = 70 for SF‐36/HADS analyses, *n* = 42 for EQ‐5D analyses). A sensitivity analysis of this analysis stratified per cohort (ONSFU and REGON cohort) can be found in Supporting Information S4. [Color figure can be viewed at wileyonlinelibrary.com]

Figure 3 shows the heatmap correlation matrix for the *baseline* analysis. The convergent validity of the CHQ was high: 9/12 hypotheses were confirmed (≤25% rejected; Table 1). *Strong* correlations were observed between the total CHQ scores and those of the HADS, SF‐36, and EQ‐5D (respectively, *ρ* = 0.68, *ρ* = −0.60 and *ρ* = −0.52). A *weak* correlation was observed between attack frequency and total CHQ score (*ρ* = 0.27). The SF‐36 Pain subscale, the most severely impacted SF‐36 subscale at baseline, correlated *strongly* with all CHQ subscales (*ρ* = −0.42 to −0.57). The EQ‐5D subscales self‐care and mobility correlated *weakly* to *negligibly* with the total CHQ score (*ρ* ≤ 0.29). A sensitivity analysis where *baseline* analysis was stratified per treatment cohort (REGON and ONSFU cohort separately) did not find any major differences caused by population heterogeneity (Table 1, Supporting Information S4).

### Step 2. Responsiveness validity

*Change* scores for the CHQ, SF‐36, EQ‐5D, and headache diaries after a 3‐month follow‐up are presented in Table 4. The mean *change* in total CHQ score was Δ −3.0 ± 18.3 (Figure 2). For non‐directional changes this was Δ8 points (IQR: 3 to 16, = irrespective of ± Δ) for the CHQ (Table 4). The attack frequency changed by a median of Δ3.6 (IQR 0.7 to 9.0; non‐directional change) attacks/week. On average, the SF‐36 total score changed Δ7.9 (IQR: 2.6 to 15.2), the EQ‐5D index Δ0.18 (IQR: 0.04 to 0.30) and the HADS total Δ4.0 (IQR: 2.0 to 7.0, Table 4).

**FIGURE 4:**
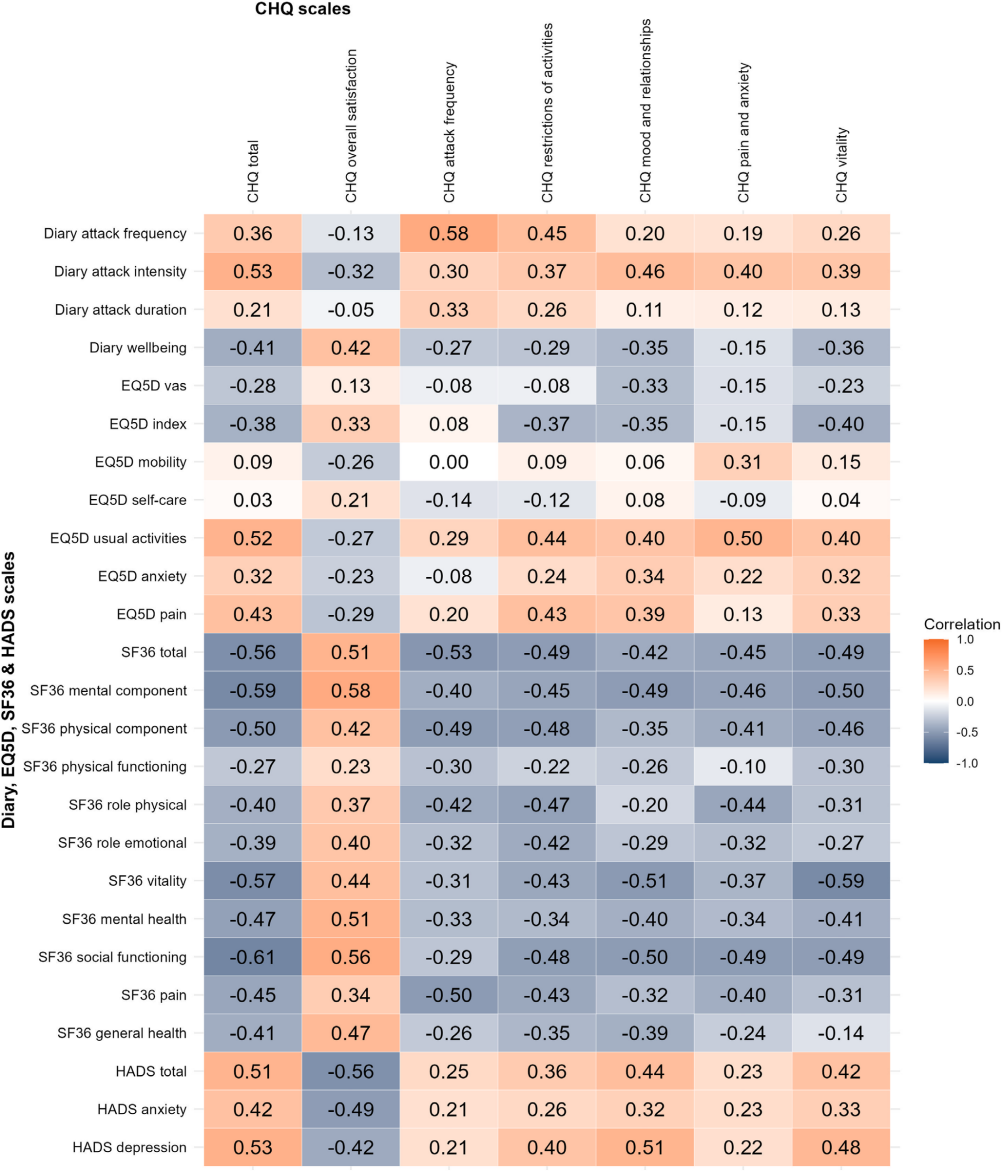
Heatmap depicting correlation coefficients of CHQ change scores with other patient reported outcomes. Heatmap depicting the Spearman correlation coefficients of the change scores of the CHQ and other QoL measurements (SF‐36, HADS, and EQ‐5D) or diary measurements. A darker color (either orange or blue) is associated with a stronger correlation. Spearman correlation interpretation: Very Strong, *ρ* ≥ 0.7; Strong, 0.4 ≤ *ρ* < 0.7; Moderate, 0.3 ≤ *ρ* < 0.4; Weak, 0.2 < *ρ* ≤ 0.3; No or negligible relationship, 0 ≤ *ρ* < 0.2. The CHQ and e‐diary were completed by all participants, the SF‐36/HADS only by the ONSFU cohort, and the EQ‐5D only by REGON participants (*N* = 82 participants in total, *n* = 48 for SF‐36/HADS analyses, *n* = 29 for EQ‐5D analyses). A sensitivity analysis of this analysis stratified per cohort (ONSFU and REGON cohort) can be found in Supporting Information S5. [Color figure can be viewed at wileyonlinelibrary.com]

Figure 4 shows the heatmap correlation matrix for the *change* analysis. The responsiveness of the CHQ was high, with all 12 predefined hypotheses confirmed (≤25% rejected; Table 2). *Strong* correlations were observed between the total change scores of the CHQ and total change scores of the HADS and the SF‐36 (respectively, *ρ* = 0.51, *ρ* = −0.56). *Moderate* correlations were observed between change scores of the EQ‐5D and CHQ (*ρ* = −0.38) and between changes in attack frequency and CHQ (*ρ* = 0.36). In general, *change* scores showed weaker correlations than *baseline* scores, except for attack frequency (*ρ* = 0.36 vs. *ρ* = 0.27). A sensitivity analysis where *change* analysis was stratified per treatment cohort (REGON and ONSFU cohort separately) did not find any major differences caused by population heterogeneity (Table 2, Supporting Information S5).

No differences were observed in secondary analyses when exploring these correlations in a poor QoL subpopulation or when using relative *change* scores (Supporting Information S6 and S7).

### Step 3. Interpretability

An MIC in the general QoL was observed in 75% (*n* = 58/77) of the population, consisting of *n* = 34 (71%) with an important change on the SF‐36 and *n* = 24 (83%) with an important change in the EQ‐5D index. Attack frequency changed with ≥30% in half of the participants (*n* = 41/82).

Three months after initial CHQ completion, QoL had improved in 47% (*n* = 36/77, *improved QoL group*), remained stable in 25% (*n* = 19/77), and deteriorated in 29% (*n* = 22/77, *deteriorated QoL group*). The total CHQ score decreased in the improved QoL group with Δ−10.9 ± 21.5, whereas in the deteriorated QoL group it increased with Δ +6.7 ± 11.4 points (Table 4). The attack frequency in the improved QoL group decreased with Δ −3.1 ± 7.7 attacks/week, whereas in the group with deteriorated QoL it increased with Δ +2.7 ± 12.8 attacks/week (Table 4).

**FIGURE 5:**
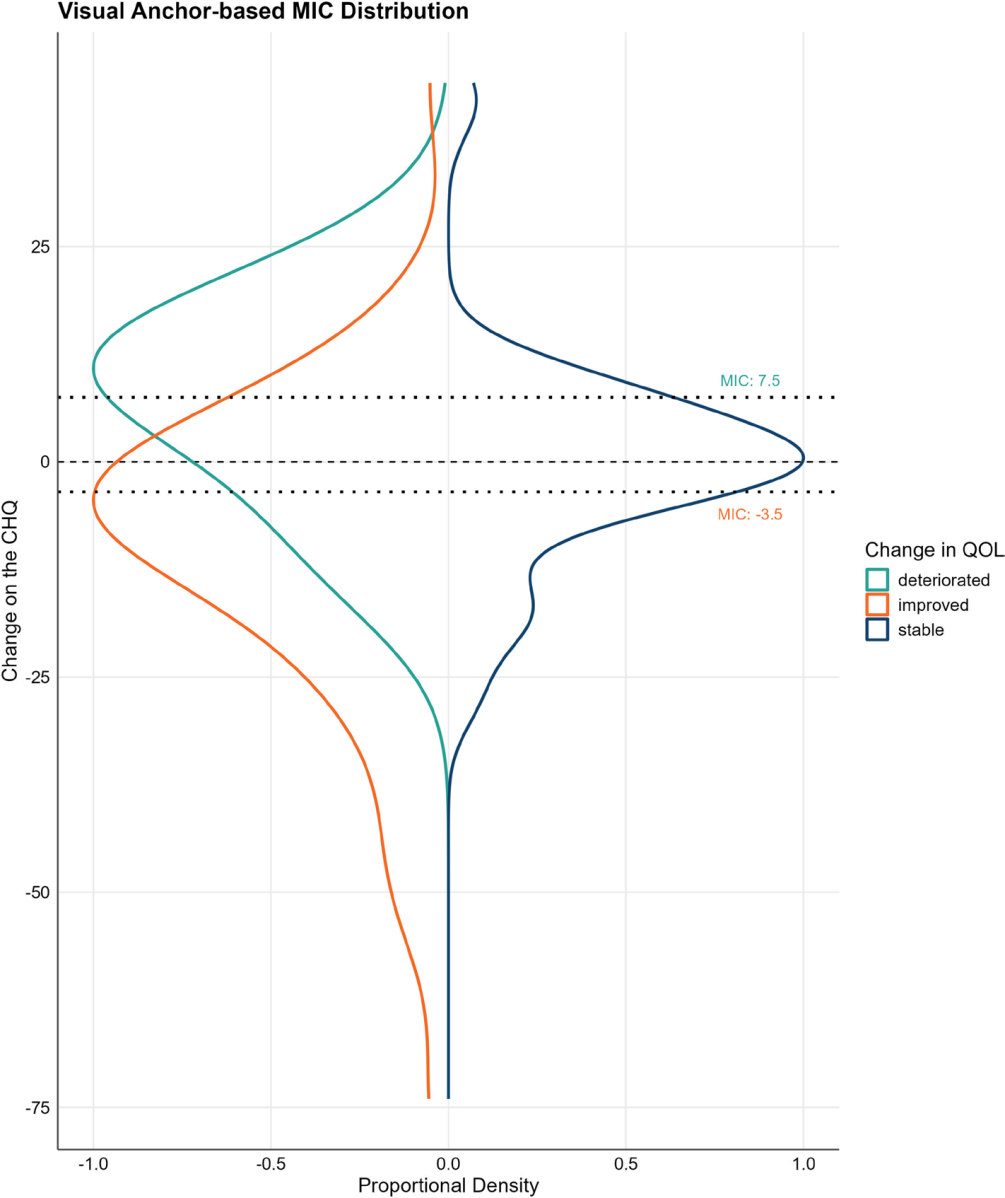
Visualization of the visual anchor‐based distribution method for MIC calculation. Visualization of the visual anchor‐based distribution method which is applied for MIC calculation. It depicts the distribution of change scores on the CHQ questionnaire of participants with an important improvement (*n* = 36, orange) or deterioration (*n* = 21, turquoise) compared with participants with no important change (=stable, *n* = 19, dark blue) on the anchors (SF‐36/EQ‐5D). The dotted lines indicate the ROC cutoff scores that are defined as MIC for an important deterioration (turquoise) or improvement (orange). MIC is defined as the optimal ROC threshold that minimizes misclassification, see Supporting Information S6 for the ROC curves. [Color figure can be viewed at wileyonlinelibrary.com]

The visual anchor‐based MIC distribution method was applied resulting in a ROC curve. For QoL *deterioration*, the MIC of the CHQ score is a minimum increase of 7.5 points. For QoL *improvement*, the MIC of the CHQ score is a minimum decrease of 3.5 points (Figure 5; Supporting Information S8).

## DISCUSSION

This prospective observational study demonstrated high convergent validity and responsiveness of the CHQ, establishing it as a reliable tool for measuring changes in QoL in individuals with cluster headache. Overall QoL in chronic cluster headache was poor, with predominant impairments attributed to pain, anxiety, depression, restrictions in daily socio‐occupational activities, and reduced vitality. Although CH symptom severity (attack frequency, duration, and intensity) correlated to QoL, other factors (e.g., mental health and activity restrictions) demonstrated stronger correlations. Finally, we provide guidance for CHQ interpretation: a decrease of 3.5 points or more in the CHQ total score is considered a minimally important improvement, whereas an increase of 7.5 points or more is considered a minimally important deterioration.

Our results confirm previous findings of a high convergent validity of the CHQ in relation to other validated QoL scales (EQ‐5D, SF‐36), and extend them by demonstrating its correlation with HADS scores and attack characteristics.^5^ Furthermore, strong correlations between the QoL summary scores (SF‐36, EQ‐5D, HADS, and CHQ) were observed.^5^ The high responsiveness of the CHQ further establishes it as a reliable tool to detect changes in QoL across multiple time‐points. Few prospective studies in CH, mostly intervention‐based, have reported QoL changes. These often showed poor correlation between clinical improvement and QoL scores.^3^ This is possibly due to a combination of weak associations between symptom severity and QoL, and a potential limited responsiveness of general QoL scales in CH. The current findings show the ability of the CHQ to address these limitations, demonstrating a strong sensitivity to changes that were previously poorly detected.

Remarkably little research has focussed on QoL in CH, despite it being one of the most painful and disabling disorders.^21^ However, the available data are in line with our findings that QoL in CH is poor compared to the general population.^3^ We found that QoL impairments are mainly linked to pain, anxiety, depression, restrictions in daily socio‐occupational activities, and reduced vitality, aligning with the themes that were noted to influence QoL in patients with successful treatment with ONS.^22^ Although attack frequency and intensity significantly affected the CHQ total score, the correlation between attack frequency and the CHQ is weak. This finding resonates with clinical anecdotes in which apparent discrepancies between attack frequency and perceived CH burden are observed for example, a greatly improved QoL with only modest reduction of symptom severity during intervention trials.^23^ This also aligns with findings of a recent study focusing on the Fear Avoidance Model (FAM) that observed a stronger correlation between CH burden and fear of attacks than with attack frequency.^24^ They proposed the FAM model as explanation: This model suggests that dysfunctional cognitive responses to actual or anticipated pain could lead to a cascade resulting in increased functional impairment. Future research is necessary to investigate whether acceptance and commitment therapy, a cognitive behavior therapy based on this model, significantly reduces cluster headache burden, as has been proven for migraine.^25^

The poor correlation between attack frequency and QoL affirms the importance of focusing on QoL in addition to symptom severity during the follow‐up of cluster headache, both in the clinic and during research. If only symptom severity or QoL is measured in clinical trials, this results in an incomplete overview of the experienced cluster headache burden and a “skewed” interpretation of patients' condition. By relying solely on attack frequency as a trial outcome, improvements in QoL can be overlooked. A non‐significant change in attack frequency may lead to a negative trial outcome. As a result, interventions that are valued by patients for reducing their burden may be incorrectly deemed “ineffective” and not implemented. During the clinical follow‐up of patients with cluster headache, particular attention should be paid to impairments caused by severe pain, anxiety, depression, and socio‐occupational impairments in addition to attack frequency. Whereas improving a patient's well‐being typically centers on reducing symptom severity through medication, it would be valuable to explore interventions directly targeting QoL, such as coping strategies. Complementary strategies targeting both disease symptoms and mental health‐related factors could more effectively reduce the overall burden of CH.

With this relatively large, well‐defined, and prospective cohort, we validated the CHQ and additionally provide a unique insight into change of QoL in cluster headache. A more comprehensive understanding of QoL was gained by comparing both QoL measures and attack characteristics and assessing data multiple time‐points. Results are strengthened by our methodology in accordance with the COSMIN guidelines. This ensured appropriate reporting and innovative approaches to report inherently subjective outcome measures with greater objectivity. By applying hypotheses‐driven correlation analyses, a clear conclusion about validity was drawn despite the absence of a gold standard for direct CHQ validation.

The study design also has some limitations, as the prospective change data were collected as part of two ongoing clinical trials in CCH. A first consequence is that the CHQ was not formally validated in ECH. It is plausible that the same QoL domains are affected in the episodic subtype, but in a less pronounced manner due to its “milder” phenotype. Previous studies that included the episodic subtype found similar validity of the CHQ across subtypes. However, since formal validation is lacking, our results should not be directly applied to the general CH population. Another limitation of the study design, is that not all patients were comparable in terms of current received treatment (ONS; ONSFU cohort; GON block or placebo, REGON cohort), different additional comparator PROMs (ONSFU cohort, HADS and SF‐36; REGON cohort, EQ‐5D), or timeline (included at baseline or during follow‐up). We attempted to address this heterogeneity by presenting data for the total population as well as for the two treatment cohorts separately and stratifying our main outcomes per treatment cohort. This stratification did not reveal major differences between groups. Our primary aim was to measure changes in quality of life irrespective of their cause. Nevertheless, it is important to note that intervention type and study context could influence these changes. Although the CHQ was administered at fixed 3‐month intervals in both studies, the timing of these assessments in relation to the intervention varied between patients. For the majority of participants, we were able to select pre and postintervention assessments to capture sufficient “change,” but this was not possible for all. Consequently, a small proportion of patients had assessments outside the preferred timeframe, which may have introduced measurement bias. Furthermore, the timeline of the responsiveness analysis was limited to a single, short‐term follow‐up period of 3 months. While sufficient to capture immediate post‐intervention changes, this timeframe does not allow evaluation of the CHQ's ability to track the more variable, long‐term course of CH, which can fluctuate substantially over months or years.

The overall sample size was adequate for the study objectives, based on recommended minima from the COSMIN guidelines, ensuring sufficient precision in the analyses. However, it is important to notice that the EQ‐5D was completed by a smaller sample size than advised by the COSMIN guidelines (*n* = 29 instead of *n* ≥ 30), as it was only completed by the REGON cohort. The EQ‐5D correlated weaker than the SF‐36 with the CHQ. This could be due to less overlap in content, as we hypothesized in our correlation hypothesis, but this could also be consequence of the small sample size.

A broader implementation of the CHQ will generate more experience on its use and observed changes. Since QoL research in CH is an emerging field, current data serve to enable implementation of the CHQ. In particular, the MIC values provide an initial framework for interpreting meaningful change, but should be viewed with caution given certain limitations. These MICs were derived according to guidelines using a combination of anchor‐ and distribution‐based methods, resulting in estimates that are both statistically and clinically meaningful. Nonetheless, some methodological limitations should be acknowledged. In line with COSMIN guidelines, we did not apply a strict minimum anchor–correlation threshold of *ρ* = 0.5; as a result, the relatively weak correlation between ΔCHQ and ΔEQ5D (*ρ* = −0.38) may have introduced noise into the responsiveness analyses. Although previous work has shown good test–retest reliability for the CHQ, our study design did not allow assessment of stability, as this requires repeating the CHQ within a short, clinically stable interval (e.g., 3 days), which was not feasible with our data.^5^, ^8^ This should be considered when interpreting small changes in CHQ scores, as we cannot yet fully distinguish true clinical change from measurement error or random variability. Lastly, because no patient‐rated global scale or external criterion was available, the clinical relevance of the MIC remains circumstantial. External validation of the CHQ against real‐world outcomes such as work performance, social relationships, or suicide risk—a key concern in this population—would be highly valuable. Our findings should therefore be regarded as a preliminary step in characterizing the CHQ's measurement properties and responsiveness rather than as a definitive clinical validation. Further studies could revisit CHQ change scores and recalibrate the MIC as larger datasets and tools for external validation become available. Ideally, the distribution of change of the CHQ would be demonstrated in a more complete (CH as well as ECH) and bigger (>200) cohort to gain more in‐depth characterization of QoL changes in CH.

## CONCLUSION

In conclusion, understanding of QoL in CH remains limited compared to the knowledge about attack frequency and intensity. We demonstrated that poor QoL in CCH is influenced by multiple factors, including mental health and activity restrictions, rather than attack frequency alone. Accordingly, clinical follow‐up of patients with CH should systematically address both attack frequency and QoL, with particular attention to impairments caused by severe pain, anxiety, depression, and restrictions in socio‐occupational activities. Although current treatment paradigms focus on indirectly improving QoL by reducing symptom severity, future research should explore interventions that directly target QoL as well, such as coping strategies. This dual approach, addressing both disease symptoms and mental health‐related factors, could more effectively reduce the overall burden of CH. The CHQ has been proven valid to measure (changes in) QoL and its implementation can lead to better insights in disease burden, enabling more targeted treatment strategies and thus improving overall disease management.

## AUTHOR CONTRIBUTIONS

**Willemijn C. Naber:** Conceptualization; investigation; writing – original draft; writing – review and editing; visualization; validation; methodology; software; project administration; formal analysis; data curation. **Paulien J. van Tilborg:** Conceptualization; writing – original draft; methodology; validation; writing – review and editing. **Roemer B. Brandt:** Conceptualization; funding acquisition; writing – original draft; methodology; validation; writing – review and editing. **Julia J. Jansen:** Data curation; writing – review and editing. **Leopoldine A. Wilbrink:** Writing – review and editing; data curation. **Wim M. Mulleners:** Writing – review and editing; data curation. **Erkan Kurt:** Writing – review and editing; data curation. **Willemijn Leen:** Writing – review and editing; data curation. **Frank J. P. M. Huygen:** Writing – review and editing; data curation. **Denise Bijlenga:** Writing – review and editing; methodology; validation. **Rolf Fronczek:** Supervision; data curation; conceptualization; investigation; funding acquisition; writing – original draft; methodology; writing – review and editing.

## FUNDING INFORMATION

Funding was received from ZonMw for the REGON study. The funders had no role in the study design, collection, analysis, and interpretation of data; in the writing of the report; and in the decision to submit the paper for publication.

## CONFLICT OF INTEREST STATEMENT

The authors report no competing interests.

**Rolf Fronczek** reports consultancy and lecture fees from Novartis, Lundbeck, AbbVie, Lilly and TEVA, and independent support from the Dutch Brain Foundation, Leiden University Fund and Innovation Fund Dutch Healthcare Providers; **Willemijn C. Naber, Paulien J. van Tilborg, Denise Bijlenga, Wim M. Mulleners, Julia J. Jansen, Willemijn Leen**, **Leopoldine A Wilbrink** **,** and **Roemer B. Brandt** report no relevant conflict of interest.

## Supporting information


**Data S1:** Supporting Information.

## Data Availability

Anonymized data not published within this article will be made available by reasonable request from any qualified investigator.
