# Supplementary material for: Quality of life changes in cluster headache: Convergent validity, responsiveness, and interpretability of the Cluster Headache Quality of Life scale as a patient‐reported outcome measure
Source: Headache. 2025 Nov 13;66(1):213–29. doi: 10.1111/head.15083 (PMC12849543; doi:10.1111/head.15083)
Supplement: Supplementary file 1 — Data S1: Supporting Information. [file HEAD-66-213-s001.docx]

# Supplemental 1 English original Cluster headache quality of life questionnaire (CHQ)

CLUSTER HEADACHE QUALITY OF LIFE QUESTIONNAIRE (CHQ)

How many times have you experienced a cluster headache attack during the last month?

Please complete the following items to indicate how often cluster headache has affected various aspects of your life DURING THE LAST MONTH or DURING YOUR MOST RECENT CLUSTER HEADACHE EPISODE

Please tick only one box for each item. Do not leave any item blank.

| **Due to cluster headache, in the past month or last episode, how often**  **have you:** | **Never** | **Occasionally** | **Sometimes** | **Often** | **Always** |
| --- | --- | --- | --- | --- | --- |
| 1. Avoided leaving the house |  |  |  |  |  |
| 2. Avoided making plans due to unpredictability of cluster headache  e.g. holidays |  |  |  |  |  |
| 3. Felt unable to complete duties at work |  |  |  |  |  |
| 4. Had difficulty in getting involved in  leisure activities e.g. cinema, theatre, etc? |  |  |  |  |  |
| 5. Avoided crowded and noisy places  e.g. public transport, pubs, etc |  |  |  |  |  |
| 6. Felt that the severity of cluster  headache affected your daily activities |  |  |  |  |  |
| 7. Been less involved in family affairs  e.g. interaction with children, planning holidays |  |  |  |  |  |
| 8. Been unable to socialise/spend time with friends and family |  |  |  |  |  |
| 9. Been unable to achieve your daily  goals and carry out routines and chores |  |  |  |  |  |
| 10. Felt less respected by others |  |  |  |  |  |
| 11. Had problems with close personal  relationship |  |  |  |  |  |
| 12. Felt you were a burden on family and friends |  |  |  |  |  |
| 13. Felt self-conscious and uncomfortable  about your appearance after a cluster headache attack (eg swelling/redness of eyes and facial sweating, etc) |  |  |  |  |  |
| 14. Felt that others are dismissive of your  cluster headaches |  |  |  |  |  |
| 15. Felt aggressive |  |  |  |  |  |
| 16. Felt bad about yourself, lost self-  confidence or felt worthless |  |  |  |  |  |
| **Due to cluster headache, in the past month or last episode, how often have you:** | **Never** | **Occasionally** | **Sometimes** | **Often** | **Always** |
| 17. Felt like harming yourself or suicidal |  |  |  |  |  |
| 18. Been irritable, impatient or less  tolerant |  |  |  |  |  |
| 19. Been forgetful e.g. missed appointments |  |  |  |  |  |
| 20. Been unable to take care of your  appearance (eg take a bath, put make- up on, change clothes, etc) |  |  |  |  |  |
| 21. Felt isolated, lonely or vulnerable |  |  |  |  |  |
| 22. Found your pain is unbearable if  untreated |  |  |  |  |  |
| 23. Dreaded that the headache would not go away |  |  |  |  |  |
| 24. Felt lacking in energy and constantly  tired |  |  |  |  |  |
| 25. Felt sleepy, worn out or less able to concentrate due to nocturnal attacks  of cluster headache |  |  |  |  |  |
| 26. Had problems concentrating e.g. reading paper, watching TV, etc |  |  |  |  |  |
| 27. Been unable to think clearly |  |  |  |  |  |
| 28. Felt tense or anxious |  |  |  |  |  |

Please rate your overall satisfaction with your life by placing a vertical line on the scale below at an appropriate point

| |

Not at all satisfied Very satisfied

# Supplemental 2: Argumentation for the hypotheses on correlation between *baseline* scores:

1. Our hypothesis is that cluster headache attacks do indirectly influence QoL, but there is no direct correlation since QoL is both influenced by objective symptoms as it is influenced by coping mechanisms. Therefore a moderate or weaker correlation is expected.
2. Both the CHQ and the HADS worse QoL is associated with higher scores. The HADS focusses mainly on the mental aspects anxiety and depression of QoL, as earlier research confirmed a higher prevalence of depression and anxiety in cluster headache we expect that this is impacted area of QoL in cluster headache and therefore has a strong or greater correlation with the HADS.
3. In the CHQ worse QoL is associated with higher scores, whereas in the SF-36 worse QoL is associated with lower scores. SF-36 and CHQ are both questionnaires focussing on different aspects of QoL and therefore a strong or greater correlation is expected.
4. In the CHQ worse QoL is associated with higher scores, whereas in the EQ-5D worse QoL is associated with lower scores. Although EQ-5D and CHQ are both questionnaires focussing on different aspects of QoL, the main focus of the EQ-5D lies on mobility and physical aspects of QoL that are less represented in the CHQ.
5. As is speculated in hypothesis 1, we expect that CHQ correlates stronger with other measurements of QoL then with an objective measurement of symptoms.
6. Since the CHQ is a cluster specific measurement and the wording of none of the subscales overlap, we expect that none of the subscales correlate very strong.
7. Since all the overall QoL question is a general question rather than a specific domain (as are the other subscales), we expect this question to correlate better with other overall QoL measurements.
8. We expect the Mood & Relationship subscale to have a big overlap with the other subscales due to a big overlap in wording and domains and the fact that it covers a big portion of the questions of the other QoL measurements. In addition the developers of the CHQ found that this subscale correlated the strongest with subscales of the SF-36 and the EQ-5D.
9. The SF-36 physical functioning subscale mainly focusses on physical limitations, since cluster headache is not a disease that directly influences mobility and occurs in otherwise relatively healthy population, we do not expect that this domain is severely impacted. In addition the CHQ does not directly focus on physical limitations, but rather on the social implications of cluster headache burden.
10. The SF-36 general health focusses mainly on the overall health, since the CHQ does not contain similar questions and the cluster headache population is (apart from CH) a relatively healthy population that in our clinical experience regards themselves as “healthy”, we expect a moderate or smaller correlation.
11. The EQ-5D mobility focusses on mobility problems, since cluster headache is not a disease that directly influences mobility and the CHQ does not directly focus on physical limitations, we expect a weak or smaller correlation.
12. The majority of the CHQ focusses on mental aspects of mental health as this is a domain that is often impaired, therefore we expect moderate or greater correlation with the SF-36 mental health subscale.

# Supplemental 3: **Argumentation for the hypotheses on correlation between *change* scores:**

Both the CHQ and the HADS worse QoL is associated with higher scores and correlate strongly at baseline, therefore we also expect a strong correlation to between both change scores.

1. In the CHQ worse QoL is associated with higher scores, whereas in the SF-36 worse QoL is associated with lower scores. They correlate strongly at baseline, therefore we also expect a strong correlation to between both change scores.
2. In the CHQ worse QoL is associated with higher scores, whereas in the EQ-5D index worse QoL is associated with lower scores. There is a moderate correlation at baseline, therefore a moderate or stronger correlation is also expected between the change scores.
3. We expect that a change in the attack frequency has a bigger impact on the QoL than the absolute attack frequency, therefore we expect a stronger correlation between the change scores of the CHQ and attack frequency than at baseline.
4. As at baseline, we expect that the change in CHQ correlates stronger with change in other measurements of QoL than with the direct symptoms, as expressed by attack frequency.

6, 10. We expect that CHQ subscales correlate weakly with other scales with topics and wording that not correspond with the CHQ scales, such as the EQ-5D subscales that focus on mobility limitations, and which had a weak correlation at baseline.

7-9, 11,12. We expect that CHQ subscales correlate stronger with other scales with similar wording and a high correlation at baseline.

# Supplemental 4: Heatmap depicting correlation coefficients of baseline CHQ scores stratified per treatment Cohort.


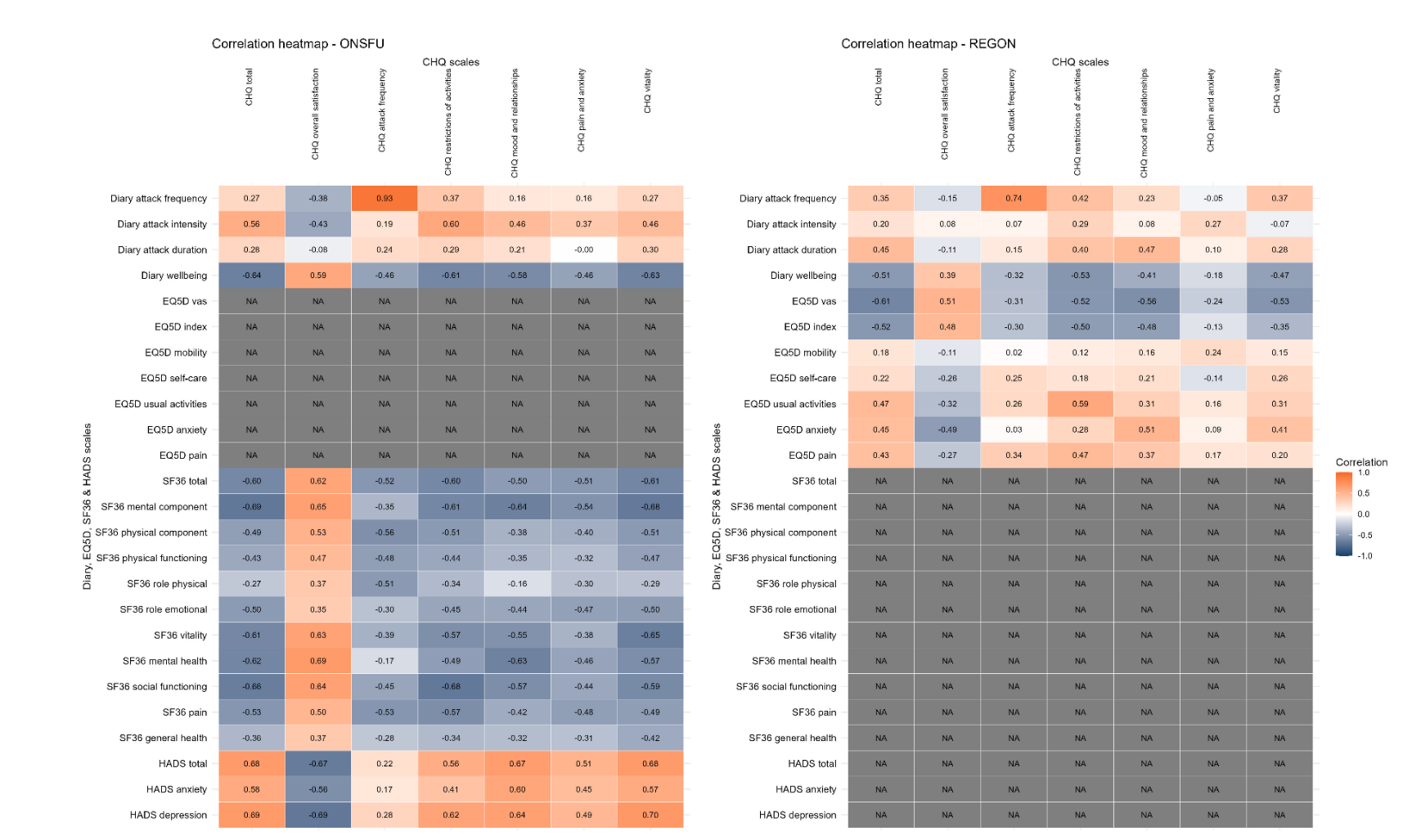


Heatmap depicting the spearman correlation coefficients of the *baseline* scores stratified per treatment cohort (ONSFU: occipital nerve stimulation, REGON: Gon injections) of the CHQ (*Cluster Headache Quality of Life scales)* and other quality of life measurements (SF-36, HADS and EQ-5D) or diary measurements. A darker colour (either orange or blue) is associated with a stronger correlation. Spearman correlation interpretation: *Very Strong*: ρ≥0.7**,** *Strong*: 0.4≤ρ<0.7, *Moderate*: 0.3≤ ρ<0.4, *Weak*: 0.2<ρ≤0.3**,** *No* or *negligible* relationship: 0≤ρ<0.2

# Supplemental 5: Heatmap depicting correlation coefficients of change CHQ scores cohort stratified per treatment cohort.


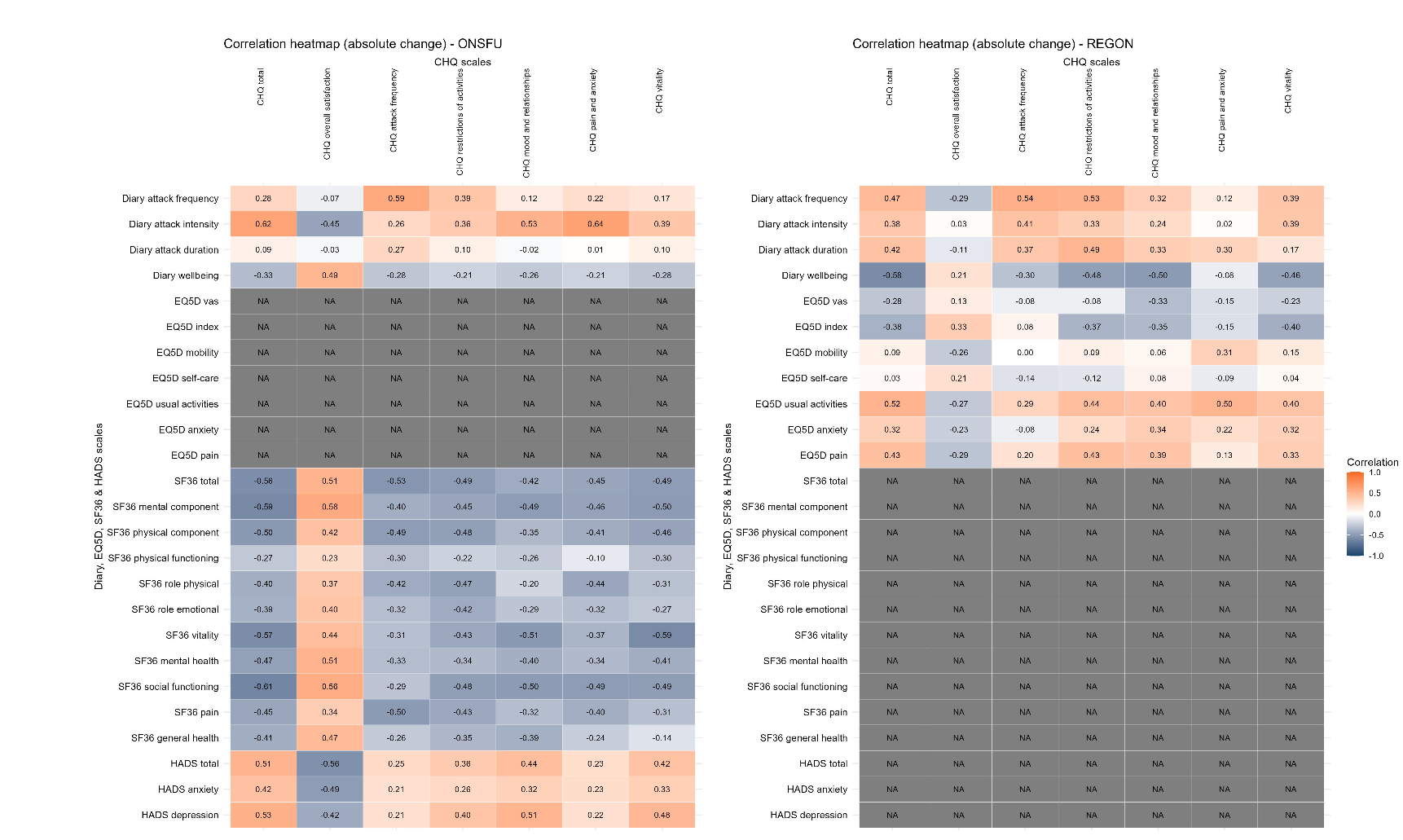


Heatmap depicting the spearman correlation coefficients of the *change* scores stratified per treatment cohort (ONSFU: occipital nerve stimulation, REGON: Gon injections) of the CHQ (*Cluster Headache Quality of Life scales)* and other quality of life measurements (SF-36, HADS and EQ-5D) or diary measurements. A darker colour (either orange or blue) is associated with a stronger correlation. Spearman correlation interpretation: *Very Strong*: ρ≥0.7**,** *Strong*: 0.4≤ρ<0.7, *Moderate*: 0.3≤ ρ<0.4, *Weak*: 0.2<ρ≤0.3**,** *No* or *negligible* relationship: 0≤ρ<0.2

# Supplemental 6: Heatmap depicting correlation coefficients of relative CHQ change scores with other patient reported outcomes.


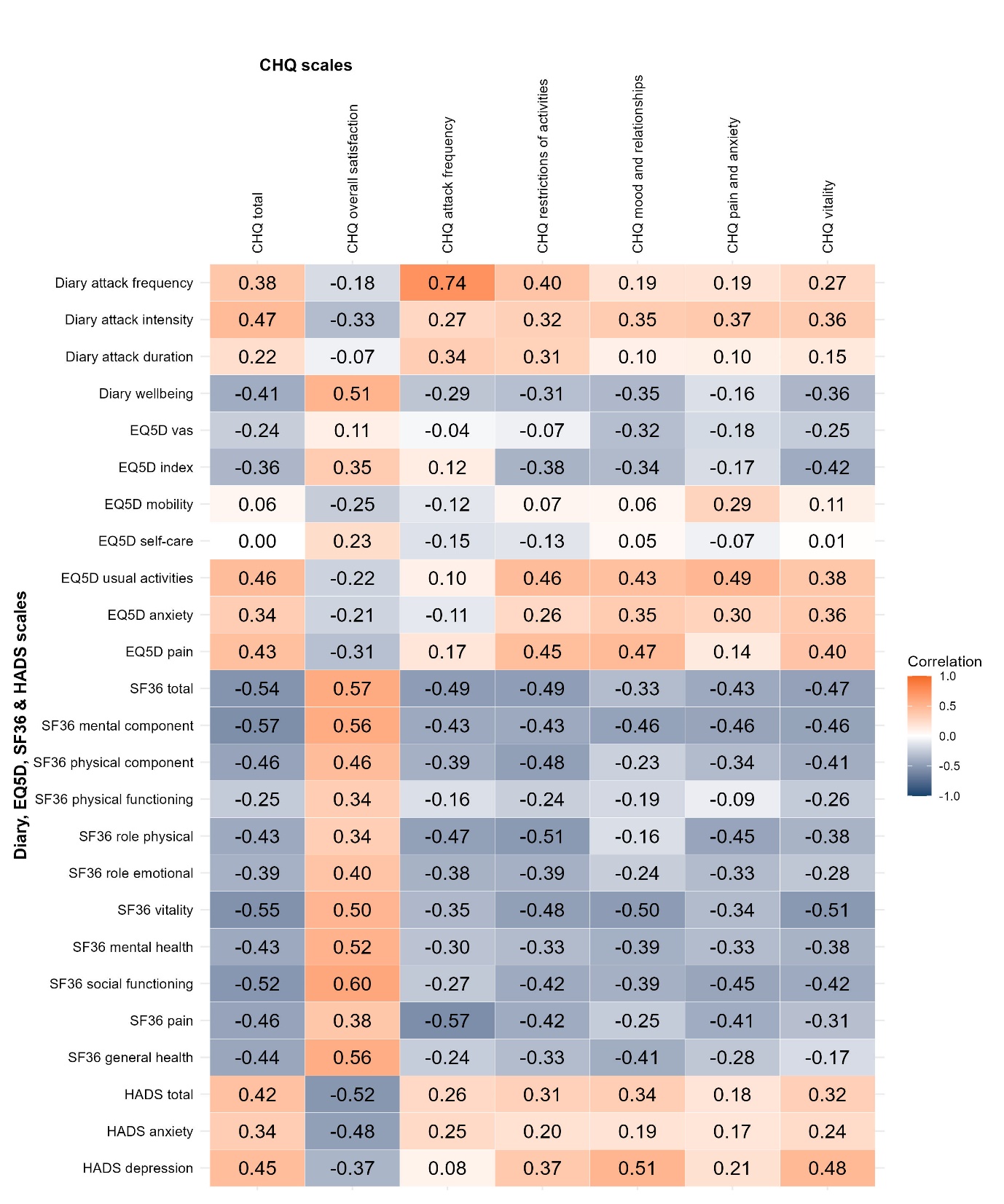


Heatmap depicting the spearman correlation coefficients of the *relative change* scores of the CHQ (*Cluster Headache Quality of Life scales)* and other quality of life measurements (SF-36, HADS and EQ-5D) or diary measurements. A darker colour (either orange or blue) is associated with a stronger correlation. Spearman correlation interpretation: *Very Strong*: ρ≥0.7**,** *Strong*: 0.4≤ρ<0.7, *Moderate*: 0.3≤ ρ<0.4, *Weak*: 0.2<ρ≤0.3**,** *No* or *negligible* relationship: 0≤ρ<0.2

# Supplemental 7: Heatmap depicting correlation coefficients of CHQ change scores with other patient reported outcomes in a poor QoL subpopulation


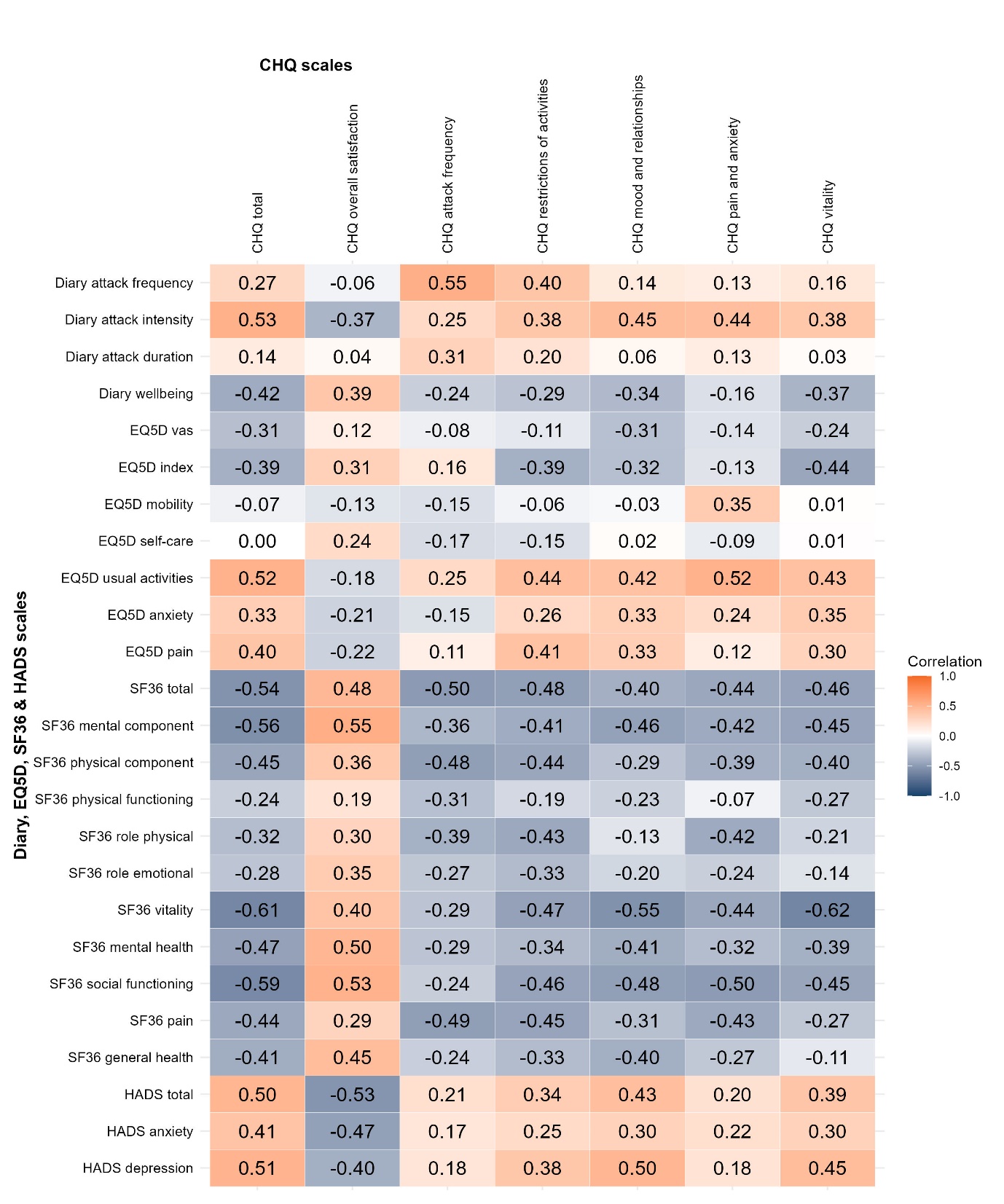


Heatmap depicting the spearman correlation coefficients of the *change* scores in a poor QoL subpopulation of the CHQ (*Cluster Headache Quality of Life scales)* and other quality of life measurements (SF-36, HADS and EQ-5D) or diary measurements. A darker colour (either orange or blue) is associated with a stronger correlation. Spearman correlation interpretation: *Very Strong*: ρ≥0.7**,** *Strong*: 0.4≤ρ<0.7, *Moderate*: 0.3≤ ρ<0.4, *Weak*: 0.2<ρ≤0.3**,** *No* or *negligible* relationship: 0≤ρ<0.2

# Supplemental 8. ROC curves to determine the optimal cut-off of the Minimally Important Change (MIC) as part of the Visual Anchor-Based Distribution method.


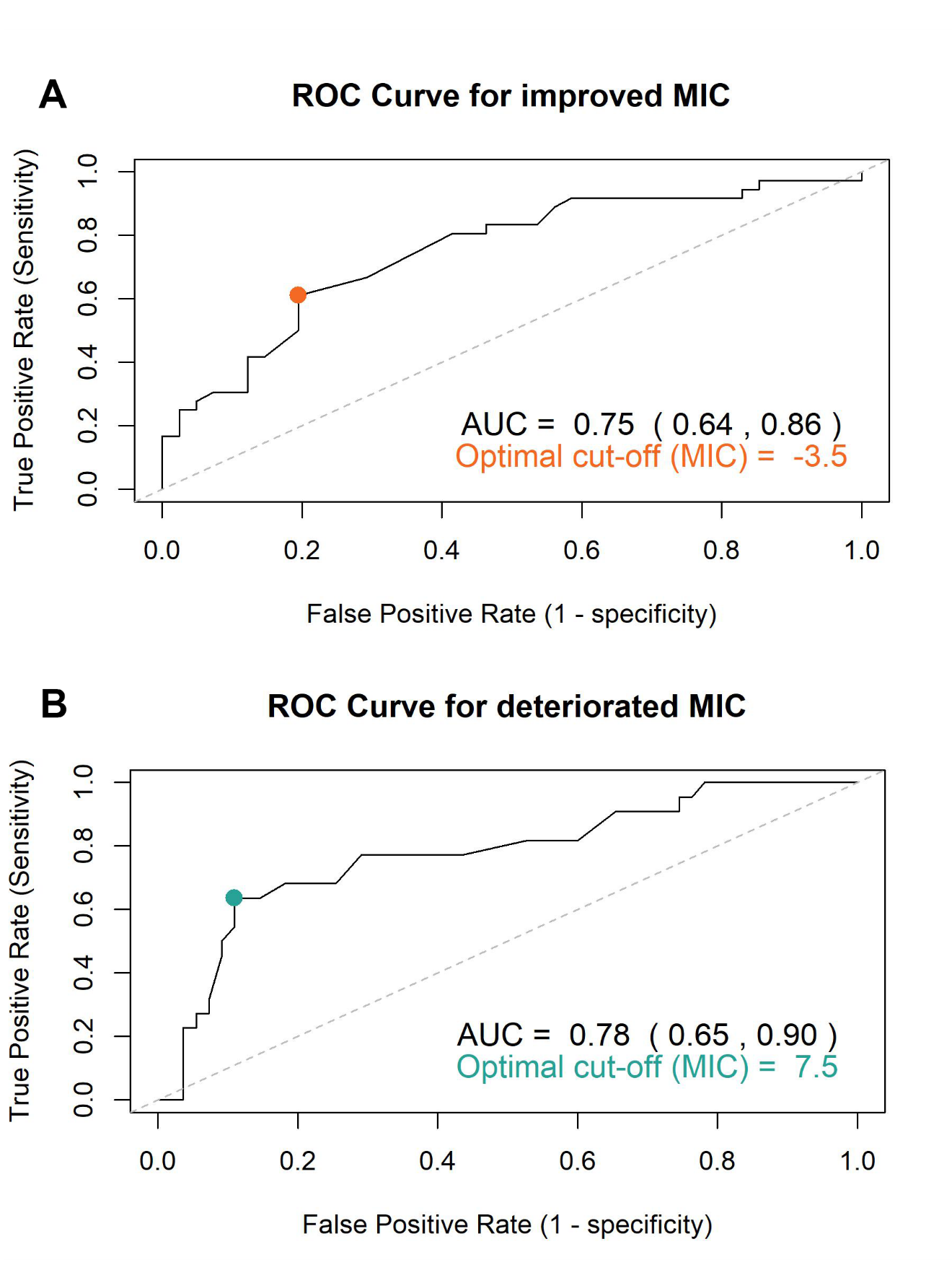
­­

ROC curves to determine the Minimally Important Change (MIC) for an important improvement (A) or deterioration (B) of the CHQ (*Cluster Headache Quality of Life scales)* . MIC is defined as the optimal ROC threshold that minimizes misclassification. AUC: Area Under the Curve.
